# Comorbidities and treatment outcomes in multidrug resistant tuberculosis: a systematic review and meta-analysis

**DOI:** 10.1038/s41598-018-23344-z

**Published:** 2018-03-21

**Authors:** Joel Philip Samuels, Aashna Sood, Jonathon R. Campbell, Faiz Ahmad Khan, James Cameron Johnston

**Affiliations:** 1 Faculty of Medicine, University of British Columbia, Vancouver, British Columbia Canada; 2 Royal College of Surgeons in Ireland, Dublin, Ireland; 3 Faculty of Pharmaceutical Sciences, University of British Columbia, Vancouver, British Columbia Canada; 4 Respiratory Epidemiology & Clinical Research Unit, Centre for Outcomes Research and Evaluation, MUHC-RI, Montreal, Quebec Canada; 5 Division of Respiratory Medicine, McGill University Health Centre, Montreal, Quebec Canada; 6 McGill International TB Centre, Montreal, Quebec Canada; 7 TB Services, BC Centre for Disease Control, Vancouver, British Columbia Canada; 8 Division of Respiratory Medicine, University of British Columbia, Vancouver, British Columbia Canada

## Abstract

Little is known about the impact of comorbidities on multidrug resistant (MDR) and extensively drug resistant (XDR) tuberculosis (TB) treatment outcomes. We aimed to examine the effect of human immunodeficiency virus (HIV), diabetes, chronic kidney disease (CKD), alcohol misuse, and smoking on MDR/XDRTB treatment outcomes. We searched MEDLINE, EMBASE, Cochrane Central Registrar and Cochrane Database of Systematic Reviews as per PRISMA guidelines. Eligible studies were identified and treatment outcome data were extracted. We performed a meta-analysis to generate a pooled relative risk (RR) for unsuccessful outcome in MDR/XDRTB treatment by co-morbidity. From 2457 studies identified, 48 reported on 18,257 participants, which were included in the final analysis. Median study population was 235 (range 60–1768). Pooled RR of unsuccessful outcome was higher in people living with HIV (RR = 1.41 [95%CI: 1.15–1.73]) and in people with alcohol misuse (RR = 1.45 [95%CI: 1.21–1.74]). Outcomes were similar in people with diabetes or in people that smoked. Data was insufficient to examine outcomes in exclusive XDRTB or CKD cohorts. In this systematic review and meta-analysis, alcohol misuse and HIV were associated with higher pooled OR of an unsuccessful outcome in MDR/XDRTB treatment. Further research is required to understand the role of comorbidities in driving unsuccessful treatment outcomes.

## Introduction

A major barrier to global tuberculosis (TB) elimination is the emergence of multidrug resistant TB (MDRTB) and extensively drug resistant TB (XDRTB)^1–3^. MDRTB is defined as resistance to at least rifampin and isoniazid^1,3,4^, while XDRTB is defined as resistance to rifampin, isoniazid, a fluoroquinolone and at least one second-line injectable agent^2,3,5^. MDR- and XDRTB require prolonged medical therapy and are associated with high rates of failure, loss to follow-up, relapse and death, largely the result of less effective and highly toxic TB treatment regimens^3^. In 2013, only 52% of MDRTB and 26% of XDRTB patients were successfully treated^6^. Understanding the drivers of unsuccessful treatment outcomes will be crucial in addressing the global MDR/XDRTB epidemic.

One driver of unsuccessful treatment outcomes may be comorbid conditions. The impact of comorbidities on drug sensitive TB treatment is well-described, with conditions such as human immunodeficiency virus (HIV) infection, diabetes mellitus (DM), chronic kidney disease (CKD) and alcohol misuse all associated with worse treatment outcomes^3,7–10^. MDR/XDRTB treatment programs often report high proportions of these comorbidities, with the prevalence of HIV, DM and alcohol misuse exceeding 10–20% in several large MDR/XDRTB cohort studies^11–15^. Unfortunately, the relationship between comorbid conditions and MDR/XDRTB treatment outcomes remains poorly described. We performed a systematic review of the published, peer-reviewed literature examining the association between specific comorbidities, including of HIV, diabetes, CKD, smoking and alcohol misuse, and MDR/XDRTB treatment outcomes^3,7,8,16–19^. We aimed to examine the relationship between comorbidities and standardized treatment outcomes including death, default, failure and a combined endpoint of unsuccessful treatment outcome.

## Methods

This systematic review and meta-analysis conforms to the Preferred Reporting Items for Systematic Reviews and Meta-Analyses (PRISMA) Guidelines^20^. Our research protocol is registered in PROSPERO (http://www.crd.york.ac.uk/PROSPERO/display_record.asp?ID=CRD42016039866, registration number CRD42016039866).

### Objectives

Our primary objective was to estimate the association between individual comorbidities and risk of unsuccessful MDR/XDRTB treatment outcome (failure, death or default, as defined below). Our secondary objectives were to estimate the association between each comorbid condition with each specific treatment outcome.

### Search strategy

Studies were identified by searching MEDLINE, EMBASE, Cochrane Central Registrar of Controlled Trials and Cochrane Database of Systematic Reviews for articles reporting MDRTB and XDRTB outcomes, published between January 1, 1980 and June 1, 2016. The full search strategy is provided in the online Appendix (Supp. Appendix). The database search was supplemented by reviewing bibliographies from all included full text articles and previous systematic reviews on MDRTB or XDRTB treatment outcomes^3,5,18,19,21–24^, as well as searching manually through all published titles from the International Journal of Tuberculosis and Lung Disease for relevant studies.

### Eligibility Criteria and Study Selection

We included studies that enrolled at least 50 participants with microbiologically-confirmed MDRTB and/or XDRTB. Eligible studies included randomized control trials (RCTs), case-control (CC), retrospective cohort (RC) and prospective cohort (PC) studies. We examined studies reported in the peer-reviewed literature in English, French and Spanish.

Studies were excluded if they reported on exclusively surgical or non-medical therapy, exclusively used standardized first-line therapy or had non-consecutive enrolment. We also excluded studies with >30% loss to follow-up, default, or treatment outcomes otherwise unaccounted for. If two studies reported duplicate data, the publication with the more detailed reports on treatment outcomes was included for meta-analysis. Studies that did not report data necessary for calculating associations between comorbidities and outcomes were excluded from the meta-analysis but their data is reported in the Appendix (Supp. Tables 7–10)^25–34^.

Two authors (JS, AS) performed the search strategy. Titles then abstracts were reviewed; studies were excluded for lack of relevance or not meeting eligibility criteria. Articles identified by either reviewer based on title and abstract were included for full text review. In full text review, any discrepancies in eligibility were resolved by a third author (JCJ).

### Treatment outcomes: definitions

Treatment outcome definitions reflected or approximated those published by Laserson *et al*.^4^.

*Cure*: completed MDRTB therapy with ≥5 negative cultures in the last 12 months of treatment; alternatively, a participant could have one positive culture followed by at least 3 negative cultures separated by 30 days with no clinical deterioration.

*Treatment completion*: completed MDRTB therapy without meeting the definition of cure.

*Death*: all-cause mortality during MDRTB therapy.

*Default*: interruption of therapy for ≥2 consecutive months for any reason.

*Treatment failure:* 2 of 5 cultures positive within the last 12 months of therapy or any culture positivity within the last 3 cultures; alternatively, failure was defined as treatment discontinuation due to lack of appropriate response or significant adverse events.

### Defining co-morbid conditions

We accepted all studies’ original criteria for defining each comorbid condition.

### Data Extraction

Data collection was performed in parallel by two authors (JS, AS), using a standardized data extraction tool, with discrepancies resolved by a third author (JCJ). Data collected included: study location, year, funding source, and design; participant characteristics (proportion with diabetes, HIV, smoking, CKD, alcohol misuse), as well as proportion with disease that was smear positive, cavitary, pulmonary, extra-pulmonary, XDRTB); treatment related variables (standardized vs individualized); and outcomes including treatment failure, default, death. When available in the original reports, effect estimates for the association between comorbidities and our outcomes of interest were extracted, along with their 95% confidence interval (95%CI).

### Data Analysis

For our primary objective, we reported the pooled relative risk (RR) of the association between each comorbidity and unsuccessful treatment (a composite of failure, death, and default). For our secondary objectives, we reported the pooled RR for the association between each comorbidity and treatment failure, death, and default, as well as with the combined outcome of death and treatment failure. Pooled RR were calculated using Mantel-Haenszel random effects meta-analysis. The I-squared statistic (*I*^2^) was used to describe heterogeneity with values less than 33% being minor/no heterogeneity, 33–66% being moderate and values greater than 66% being significant heterogeneity. Outcomes were a composite of MDRTB and XDRTB data.

Statistical analyses were performed using Review Manager Software from the Cochrane Group. (*Review Manager (RevMan) [Computer Program] Version 5.3. Copenhagen: The Nordic Cochrane Centre, The Cochrane Collaboration Group, 2014*.)

### Assessment of quality and bias

Study quality was assessed using the Newcastle-Ottawa Scale (NOS) for cohort studies^35^. Publication bias was assessed using visual inspection of funnel plots (Supp. Figs 1–4).

**Figure 1:**
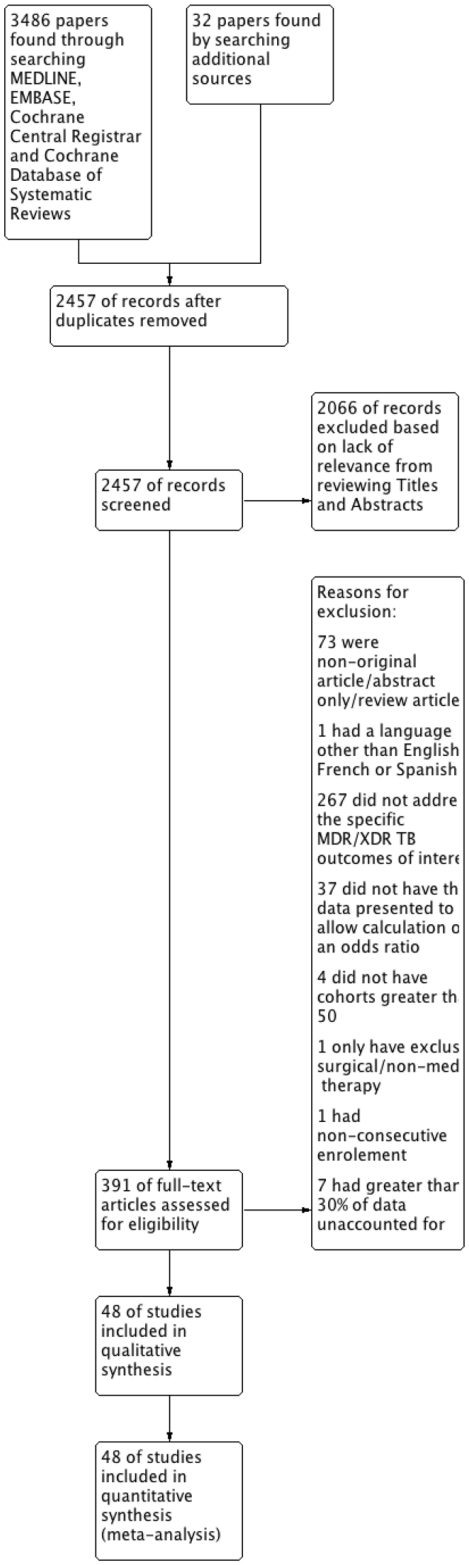
Flowchart showing Selection Process for Included Articles.

**Figure 2:**
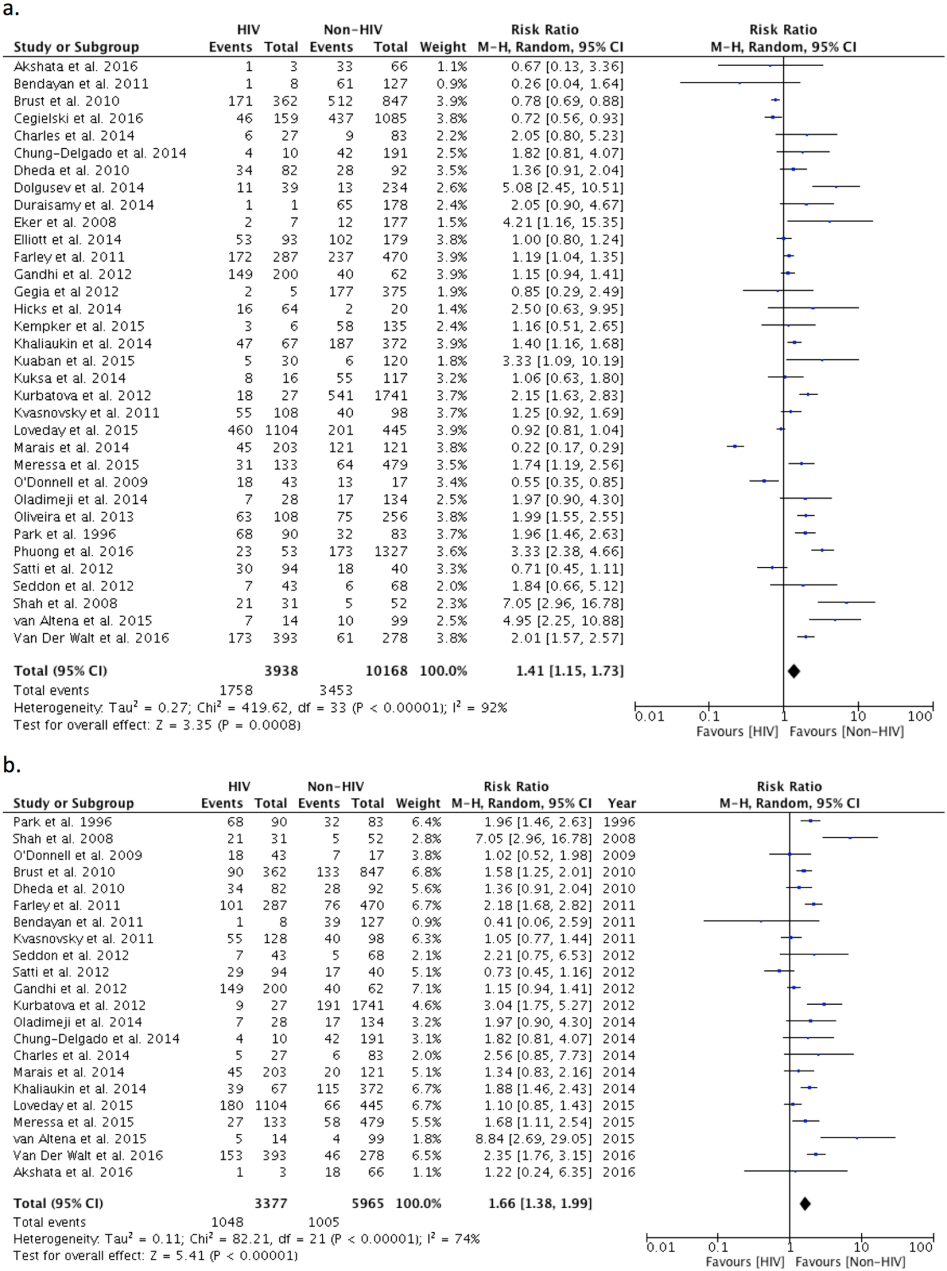
(**a**) Relative Risk of primary outcome in MDR/XDRTB patients living with HIV compared to those without HIV infection. (**b**) Relative Risk of mortality in MDR/XDRTB patients living with HIV compared to those without HIV infection.

**Figure 3:**
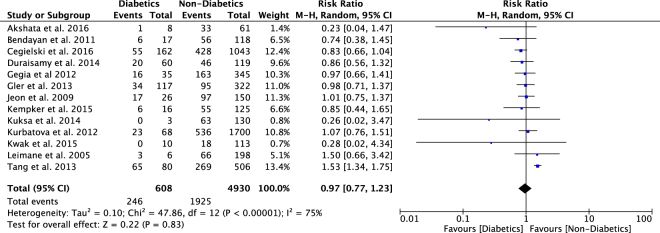
Relative Risk of primary outcome in MDR/XDRTB patients with DM compared to those without DM.

**Figure 4:**
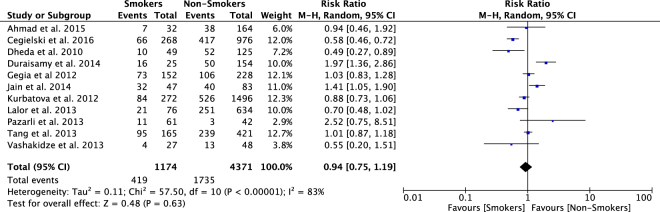
Relative Risk of primary outcome in MDR/XDRTB patients that smoke compared to non-smokers.

## Results

Our literature search yielded 2457 titles; 2066 were excluded based on review of title and abstract, leaving 391 articles for full text review (Fig. 1). After full text review, 55 articles were eligible for analysis, with 7 articles reporting ≥30% of default, transfer out and loss to follow-up^36–42^, leaving 48 papers with 18,257 participants for final analysis^10–15,28,34,43–82^. There were no overlapping study populations in the final review.

All 48 included studies were published between 1996 and 2016 with a median population of 235 (range 60–1768) (Tables 1 and 2). The majority (71.4%) of studies were retrospective cohorts^10–13,15,28,34,44,46,48–50,54–57,59–61,63,65–71,73,74,76–79,81,82^, while 26.5% of studies were prospective cohorts^14,43,45,47,51–53,58,62,64,75,80^ and one study was case-control in design^72^. There were no relevant RCTs that met our inclusion criteria. None of the included studies received direct funding from pharmaceutical companies.

**Table 1:** Characteristics of Included Studies.

Study	Country	Study Design	Quality (NOS)	Sample Size (n)	Age (median/IQRMean/SD)	Male n(%)	Smear Positive n(%)	Cavitary Disease n(%)	XDR-TB n(%)	Length of Treatment (median/IQR)
Meressa *et al*.^43^	Ethiopia	PC	8	612	27 (22–36)	325 (0.53)	N/A	306 (0.5)	N/A	N/A
Kempker *et al*.^44^	Georgia	RC	5	141	34 (27–46)	103 (0.73)	46 (0.33)	30 (0.21)	9 (0.06)	N/A
Gegia *et al*.^45^	Georgia	PC	7	380	38 (16–81)	271 (0.71)	N/A	N/A	N/A	>18 months
Miller *et al*.^46^	Russia	RC	9	407	N/A	N/A	N/A	N/A	20 (0.05)	N/A
Kwak *et al*.^10^	South Korea	RC	7	123	37 (27–56)	69 (0.56)	N/A	85 (0.69)	26 (0.21)	24.4 months (18.4–27.3)
Leimane *et al*.^28^	Latvia	RC	6	204	N/A	153 (0.75)	90 (0.44)	148 (0.73)	N/A	N/A
Loveday *et al*.^47^	South Africa	PC	9	1549	N/A	746 (0.48)	1048 (0.68)	N/A	N/A	N/A
Seddon *et al*.^11^	South Africa	RC	9	111	50 (19–108)	46 (0.41)	53 (0.48)	38 (0.34)	5 (0.05)	18 months
Duraisamy *et al*.^12^	India	RC	7	179	N/A	139 (0.78)	N/A	94 (0.53)	N/A	N/A
Dheda *et al*.^13^	South Africa	RC	9	174	N/A	85 (0.49)	N/A	N/A	174 (1)	6.9 months (3–12)
Kvasnovsky *et al*.^48^	South Africa	RC	7	206	N/A	101 (0.49)	89 (0.43)	50 (0.24)	206 (1)	N/A
Shah *et al*.^49^	USA	RC	9	83	N/A	53 (0.64)	57 (0.69)	24 (0.29)	83 (1)	N/A
Elliott *et al*.^50^	South Africa	RC	9	272	N/A	135 (0.50)	131 (0.48)	N/A	27 (0.10)	N/A
Vashakidze *et al*.^51^	Georgia	PC	8	75	30 (15–54)	47 (0.63)	12 (0.16)	71 (0.95)	24 (0.32)	N/A
Kuaban *et al*.^52^	Cameroon	PC	6	150	N/A	77 (0.51)	N/A	N/A	N/A	368 days (363–375)
Brust *et al*.^53^	South Africa	PC	7	1209	33(26–41)	737 (0.61)	N/A	N/A	N/A	N/A
Van Altena *et al*.^54^	Netherlands	RC	9	113	N/A	69 (0.61)	56 (0.50)	N/A	4 (0.04)	445 days
O’Donnell *et al*.^55^	South Africa	RC	7	60	N/A	26 (0.43)	N/A	N/A	60 (1)	N/A
Oladimeji *et al*.^56^	Nigeria	RC	6	162	N/A	105 (0.65)	N/A	N/A	N/A	N/A
Hicks *et al*.^57^	South Africa	RC	7	84	8 (4–12)	36 (0.43)	40 (0.48)	N/A	6 (0.07)	644 days (559–728)
Ahmad *et al*.^58^	Pakistan	RC/PC	8	196	31.5 (16.8–46.2)	92 (0.47)	178 (0.91)	68 (0.35)	N/A	24 months (20–34)
Phuong *et al*.^59^	Vietnam	RC	9	1380	N/A	1074 (0.78)	1167 (0.85)	N/A	N/A	N/A
Akshata *et al*.^82^	India	RC	4	69	N/A	46 (0.67)	69 (1)	34 (0.49)	2 (0.03)	N/A
Kuksa *et al*.^60^	Latvia	RC	9	133	N/A	86 (0.65)	79 (0.59)	90 (0.68)	133 (1)	N/A
Eker *et al*.^61^	Germany	RC	8	184	N/A	139 (0.76)	N/A	N/A	7 (0.04)	N/A
Cox *et al*.^62^	Uzbekistan	PC	7	87	34 (17–72)	53 (0.61)	N/A	61 (0.70)	N/A	22 months (18–30)
Cegielski *et al*.^14^	Multiple*	PC	9	1244	N/A	771 (0.62)	1034 (0.83)	750 (0.60)	N/A	651 days (589–736)
Bendayan *et al*.^63^	Israel	RC	7	135	40	102 (0.76)	93 (0.69)	N/A	11 (0.08)	N/A
Park *et al*.^15^	USA	RC	7	173	40	159 (0.92)	94 (0.54)	54 (0.31)	N/A	N/A
Farley *et al*.^64^	South Africa	PC	9	757	N/A	448 (0.59)	434 (0.57)	N/A	N/A	N/A
Satti *et al*.^65^	South Africa	RC	9	134	N/A	79 (0.59)	15 (0.11)	96 (0.72)	N/A	22.9 monhts (21.6–24)
Dolgusev *et al*.^66^	Moldova	RC	9	273	N/A	N/A	N/A	N/A	N/A	N/A
Khaliaukin *et al*.^67^	Belarus	RC	9	439	N/A	367(0.84)	N/A	N/A	N/A	N/A
Kurbatova *et al*.^68^	Multiple^¶^	RC	5	1768	36	1237 (0.70)	N/A	1085 (0.61)	57 (0.03)	21 months (17–25)
Oliveira *et al*.^69^	Portugal	RC	9	364	N/A	259 (0.71)	N/A	157 (0.43)	107 (0.29)	N/A
Franke *et al*.^70^	Peru	RC	8	671	31.4 (19.3–43.5)	408 (0.61)	N/A	N/A	N/A	N/A
Lalor *et al*.^71^	Uzbekistan	RC	7	710	29.4 (23.1–40.3)	343 (0.48)	N/A	N/A	N/A	N/A
Gandhi *et al*.^72^	South Africa	cc	6	262	34 (29–43)	70 (0.27)	161 (0.61)	N/A	139 (0.53)	N/A
Tang *et al*.^34^	China	RC	9	586	N/A	395 (0.67)	N/A	389 (0.66)	169 (0.29)	N/A
Jeon *et al*.^73^	South Korea	RC	7	176	42.1 (28–56.2)	127 (0.72)	N/A	132 (0.75)	176 (1)	N/A
Pazarli *et al*.^74^	Turkey	RC	6	103	40.5 (27–54)	81 (0.79)	N/A	N/A	N/A	N/A
Jain *et al*.^75^	India	PC	8	130	N/A	81 (0.62)	N/A	N/A	N/A	N/A
Charles *et al*.^76^	Haiti	RC	7	110	28 (23–37)	50 (0.45)	N/A	N/A	N/A	22.5 months (11.5–31)
Shin *et a l*.^77^	Russia	RC	8	244	32.3 (16–65)	211(0.86)	N/A	N/A	N/A	N/A
Marais *et al*.^78^	South Africa	RC	7	324	N/A	170 (0.52)	N/A	N/A	N/A	N/A
Van Der Walt *et al*.^79^	South Africa	RC	8	671	N/A	N/A	N/A	N/A	N/A	N/A
Gler *et al*.^80^	Philippines	RC/PC	8	439	N/A	271 (0.62)	N/A	216 (0.49)	N/A	N/A
Chung-Delgado *et al*.^81^	Peru	RC	7	201	N/A	127 (0.63)	151 (0.75)	N/A	N/A	N/A

^*^Estonia, Latvia, Philippines, Peru, Russia, South Africa, South Korea, Taiwan, Thailand; ^¶^Estonia, Lativa, Philippines, Peru, Russia.

**Table 2:** Overall Treatment outcomes.

Study	DM n(%)	HIV n(%)	Smoker n(%)	ETOH n(%)	Regimen n(%)*	Treatment Success n(%)	Default, Death, Failure (%)	Data unknown (%)^¶^
Meressa *et al*.^43^	33 (0.05)	133 (0.21)	67 (0.11)	N/A	S	481 (0.79)	95 (0.15)	0.06
Kempker *et al*.^44^	16 (0.10)	6 (0.04)	N/A	N/A	I	79 (0.5)	N/A	N/A
Gegia *et al*.^45^	35 (0.09)	5 (0.01)	152 (0.4)	94 (0.24)	I	201 (0.53)	179 (0.47)	0.22
Miller *et al*.^46^	N/A	N/A	343 (0.84)	253 (0.62)	N/A	247 (0.61)	160 (0.39)	0.23
Kwak *et al*.^10^	10 (0.08)	N/A	38 (0.31)	N/A	I	103 (0.83)	18 (0.15)	0.05
Leimane *et al*.^28^	6 (0.03)	1 (0.005)	N/A	125 (0.61)	N/A	135 (0.66)	69 (0.34)	0.13
Loveday *et al*.^47^	N/A	1104 (0.71)	N/A	N/A	S	866 (0.56)	661 (0.43)	0.23
Seddon *et al*.^11^	N/A	43 (0.39)	N/A	N/A	I	82 (0.74)	14 (0.13)	0.14
Duraisamy *et al*.^12^	60 (0.33)	1 (0.006)	25 (0.14)	16 (0.09)	S	112 (0.63)	66 (0.37)	0.15
Dheda *et al*.^13^	N/A	82 (0.47)	49(0.28)	N/A	S/I	N/A	62 (0.36)	N/A
Kvasnovsky *et al*.^48^	N/A	108(0.52)	N/A	N/A	I	N/A	95 (0.46)	N/A
Shah *et al*.^49^	N/A	31 (0.37)	N/A	N/A	N/A	33 (0.40)	26 (0.31)	0.28
Elliott *et al*.^50^	N/A	93 (0.34)	N/A	N/A	N/A	113 (0.42)	155 (0.57)	N/A
Vashakidze *et al*.^51^	6 (0.08)	1 (0.01)	27 (0.36)	11 (0.15)	I	59 (0.79)	13 (0.17)	0.16
Kuaban *et al*.^52^	N/A	30 (0.2)	N/A	N/A	S	N/A	N/A	0.03
Brust *et al*.^53^	N/A	362 (0.30)	N/A	N/A	S	526 (0.44)	683 (0.56)	0.21
Van Altena *et al*.^54^	N/A	14 (0.12)	N/A	N/A	N/A	89 (0.79)	17 (0.15)	0.13
O’Donnell *et al*.^55^	N/A	43 (0.72)	N/A	N/A	N/A	N/A	31 (0.52)	0.10
Oladimeji *et al*.^56^	N/A	28 (0.17)	N/A	N/A	S	N/A	24 (0.15)	N/A
Hicks *et al*.^57^	N/A	64 (0.76)	N/A	N/A	N/A	66 (0.79)	18 (0.21)	0.06
Ahmad *et al*.^58^	17 (0.09)	N/A	32 (0.16)	N/A	I	136 (0.69)	45 (0.23)	0.04
Phuong *et al*.^59^	N/A	53 (0.04)	N/A	N/A	S	1008 (0.73)	196 (0.41)	0.13
Akshata *et al*.^82^	8 (0.12)	3 (0.04)	N/A	N/A	S	33 (0.48)	34 (0.49)	0.17
Kuksa *et al*.^60^	3 (0.02)	16 (0.12)	N/A	64 (0.48)	I	70 (0.53)	63 (0.47)	0.12
Eker *et al*.^61^	N/A	7 (0.04)	N/A	N/A	N/A	109 (0.59)	17 (0.09)	0.14
Cox *et al*.^62^	N/A	N/A	27 (0.31)	20 (0.23)	S/I	54 (0.62)	33 (0.38)	0.14
Cegielski *et al*.^14^	162 (0.13)	159 (0.13)	268 (0.22)	179 (0.14)	S/I	722 (0.58)	483 (0.39)	0.22
Bendayan *et al*.^63^	17 (0.13)	8 (0.06)	N/A	34 (0.25)	I	70 (0.52)	62 (0.46)	0.09
Park *et al*.^15^	N/A	90 (0.52)	N/A	N/A	N/A	N/A	100 (0.58)	0.18
Farley *et al*.^64^	N/A	287 (0.38)	N/A	N/A	S	348 (0.46)	409 (0.54)	0.21
Satti *et al*.^65^	N/A	94 (0.70)	N/A	N/A	S	83 (0.62)	48 (0.36)	0.03
Dolgusev *et al*.^66^	N/A	39 (0.14)	N/A	N/A	I	122 (0.45)	95 (0.35)	0.21
Khaliaukin *et al*.^67^	N/A	67 (0.15)	N/A	N/A	I	148 (0.33)	234 (0.53)	0.13
Kurbatova *et al*.^68^	68 (0.04)	27 (0.015)	272 (0.15)	489 (0.28)	I	1156 (0.65)	559 (0.32)	0.17
Oliveira *et al*.^69^	N/A	108 (0.30)	N/A	61 (0.17)	N/A	226 (0.62)	138 (0.38)	0.07
Franke *et al*.^70^	N/A	N/A	N/A	28 (0.04)	I	N/A	67 (0.10)	0.10
Lalor *et al*.^71^	N/A	N/A	76 (0.11)	90 (0.13)	I	438 (0.62)	272 (0.38)	0.20
Gandhi *et al*.^72^	N/A	200 (0.76)	N/A	N/A	S	N/A	189 (0.72)	N/A
Tang *et al*.^34^	80 (0.14)	N/A	165 (0.28)	N/A	I	240 (0.41)	334 (0.57)	0.08
Jeon *et al*.^73^	26 (0.15)	N/A	N/A	N/A	I	28 (0.16)	114 (0.65)	0.28
Pazarli *et al*.^74^	17 (0.17)	N/A	61 (0.59)	N/A	I	89 (0.86)	14 (0.14)	0.02
Jain *et al*.^75^	N/A	N/A	46 (0.35)	27 (0.21)	S	58 (0.45)	72 (0.55)	0.23
Charles *et al*.^76^	N/A	27 (0.25)	N/A	N/A	N/A	43 (0.39)	15 (0.14)	0.04
Shin *et al*.^77^	9 (0.04)	N/A	215 (0.88)	86 (0.35)	I	188 (0.77)	56 (0.23)	0.11
Marais *et al*.^78^	N/A	203 (0.58)	N/A	N/A	S/I	158 (0.45)	166 (0.47)	0.29
Van Der Walt *et al*.^79^	N/A	393 (0.59)	N/A	N/A	S	404 (0.60)	234 (0.35)	0.09
Gler *et al*.^80^	117 (0.27)	N/A	N/A	N/A	I	310 (0.71)	129 (0.29)	0.18
Chung-Delgado *et al*.^81^	N/A	10 (0.05)	N/A	N/A	S/I	155 (0.77)	46(0.23)	N/A

*Default/Loss to follow up/data unavailable/Transfer Out; ^¶^I = individualized; S = standardized.

^§^Treatment Completion and/or cure.

### Outcomes in people living with HIV

There were 34 studies with 14,106 participants reporting outcomes in people living with HIV (PLWH) compared with people without HIV infection (Fig. 2a)^11–15,43–45,47–50,52–57,59–61,63–69,72,76,78,79,81,82^. The proportion of study participants that were PLWH varied from 0.6 to 76%. The pooled RR for unsuccessful outcome in PLWH compared to those without HIV infection was 1.41 (95%CI: 1.15–1.73). Heterogeneity was significant (*I*^2^ = 92%, p < 0.001); funnel plots were not consistent with publication bias (Supp. Fig. 1).

We were able to analyze the outcome of mortality in 22 studies in 9342 PLWH (Fig. 2b)^11,13,15,43,47–49,53–56,63–65,67,68,72,76,78,79,81,82^; pooled RR for mortality in PLWH was 1.66 (95%CI: 1.38–1.99; *I*^2^ = 74%, p < 0.001). Treatment default was reported in 9 studies with 6311 participants; pooled RR for default was 1.05 (95%CI: 0.82–1.35; *I*^2^ = 52%, p = 0.04) (Supp. Fig. 8)^47,53,54,64,65,68,76,79^. There were 7 studies with 5930 participants reporting on treatment failure; pooled RR for treatment failure was 0.75 (95%CI: 0.44–1.29; *I*^2^ = 55%, p = 0.04) (Supp. Fig. 9)^43,47,64,65,67,68,79^. Finally, there were 28 studies with 12,999 participants compared the combined outcome of death and treatment failure with a pooled RR of 1.61 in PLWH (95% CI: 1.32–1.96; *I*^2^ = 86%, p < 0.00001) (Supp. Fig. 10)^11–15,43,45,47–49,53–56,59,60,63–65,67–69,72,76,78,79,81,82^.

We examined forest plots by study year, study quality, regional gross domestic product (GDP) and proportion of people using antiretroviral therapy (ART) (Supp. Figs 11–14). There was no obvious trend on visual inspection when comparing studies by year of publication or study quality. There was a greater effect of HIV on unsuccessful treatment outcomes in low-income regions (RR 2.23; 95%CI: 1.60–3.11) compared with high income regions (RR 1.22; 95%CI 0.97–1.53). On inspection of forest plots by stratified by ART usage, there was no clear visual trend towards improved outcomes amongst those with the highest proportion of ART usage (Supp. Fig. 14 and Supp. Table 5). Additionally there was only one study that reported outcomes according to whether or not participants were on ARTs^67^. Between-study heterogeneity did not decrease in any stratified analyses with the exception of study heterogeneity being reduced amongst in PLWH in low GDP countries (*I*^2^ = 41%, p = 0.12) (Supp. Figs 8–11). Unfortunately, data available to us was insufficient to perform meta-regression.

### Outcomes in participants with diabetes

There were 13 studies with 5538 participants reported unsuccessful treatment outcomes in people with diabetes compared to people without diabetes^10,12,14,28,34,44,45,60,63,68,73,80,82^. The pooled RR for unsuccessful outcome was 0.97 (95%CI: 0.77–1.23) (Fig. 3), with significant heterogeneity observed (*I*^2^ = 75%, p < 0.001). Funnel plot inspection suggested some potential for publication bias with smaller studies demonstrating negative outcomes amongst those with diabetes (Supp. Fig. 2).

Analyses of secondary outcomes were not feasible due to insufficient data; three studies reported data relevant for mortality^63,68,82^, one study reported default^37^ and one study reported treatment failure^68^. Further analysis by GDP and study quality did not significantly change outcomes. Heterogeneity was reduced when low GDP countries were analyzed (*I*^2^ = 19%, p = 0.29) although only three studies reported data from low GDP countries^12,80,82^ (Supp. Figs 5–7). Unfortunately, data available to us was insufficient to perform meta-regression.

### Outcomes in Smokers

There were 11 studies with 5545 participants reporting the primary outcome in smokers versus non-smokers^12–14,34,45,51,58,68,71,74,75^. The pooled RR for unsuccessful outcome was 0.94 (95%CI: 0.75–1.19) (Fig. 4). Heterogeneity was significant (*I*^2^ = 83%, p < 0.001), and no publication bias was noted on visual inspection of funnel plots (Supp. Fig. 3).

Analyses of secondary outcomes was not feasible due to insufficient data, with only two studies reporting data relevant for mortality^13,68^, two studies reporting default^68,71^ and two reporting failure^68,83^. There was a visual trend towards improved outcomes in smokers in higher quality studies (Supp. Fig. 15). Analysis of the primary outcome according to regional GDP revealed a decrease in heterogeneity amongst high GDP countries (Supp. Figs 16–17). Unfortunately, data available to us was insufficient to perform meta-regression.

### Outcomes in participants with Alcohol Misuse

**Figure 5:**
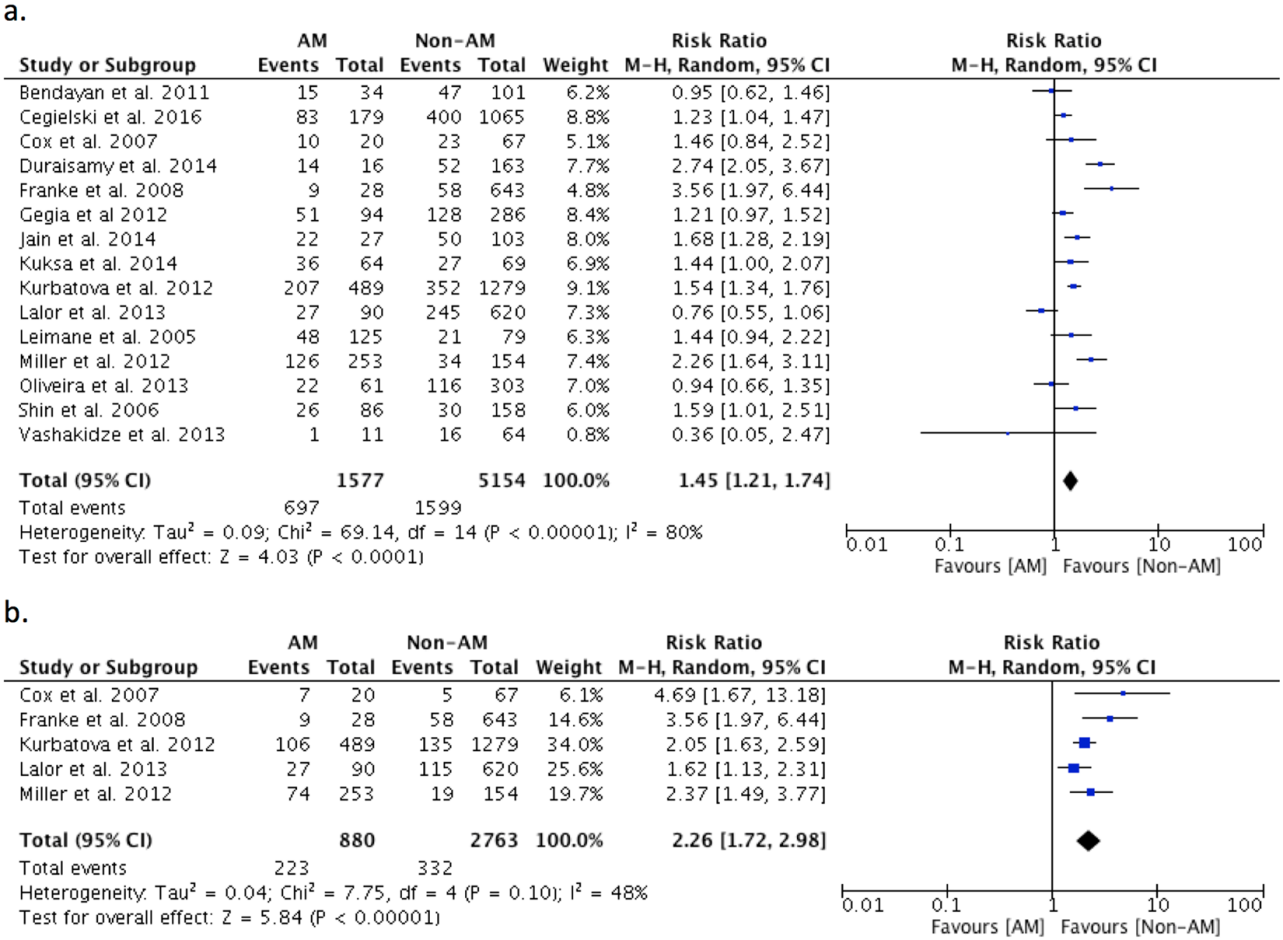
(**a**) Relative Risk of primary outcome in MDR/XDRTB patients with alcohol misuse (AM) compared to those without alcohol misuse (Non-AM). (**b**) Relative Risk of default in MDR/XDRTB patients with alcohol misuse (AM) compared to those without alcohol misuse (Non-AM).

There were 15 studies with 6731 participants reporting on the primary outcome in people with alcohol misuse compared to those without alcohol misuse^12,14,28,45,46,51,60,62,63,68–71,75,77^. The pooled RR for unsuccessful treatment outcome was 1.45 (95%CI: 1.21–1.74) (Fig. 5a) with significant heterogeneity (*I*^2^ = 80%, p < 0.001). There was no detectable publication bias (Supp. Fig. 4).

Five studies^46,62,68,70,71^ with 3643 participants enabled comparison of treatment default. The pooled RR for default was 2.26 in people with alcohol misuse (95%CI: 1.72–2.98) (Fig. 5b), with moderate heterogeneity (*I*^2^ = 48%, p = 0.10). Additional analyses of secondary outcomes were not feasible due to insufficient data with three studies reporting relevant data for mortality^46,63,68^, and two studies reporting on treatment failure^46,68^. There was no visual trend or change in heterogeneity observed when stratifying by study quality or country GDP (Supp. Figs 18–20). Unfortunately, data available to us was insufficient to perform meta-regression.

### Chronic Kidney Disease Outcomes

Only two studies^10,84^ reported outcomes in participants with MDR/XDRTB and CKD. Analysis of primary and secondary outcomes was not possible due to insufficient data.

## Discussion

In this systematic review examining the association between comorbidities and MDR/XDRTB treatment outcomes, we found that both HIV and alcohol misuse were associated with an increased pooled relative risk of unsuccessful treatment outcome in MDRTB patients. We found no clear association between unsuccessful treatment outcome with the comorbidities of smoking, diabetes or CKD.

To our knowledge, this is the first systematic review to comprehensively examine the relationship between comorbidities and MDR/XDRTB treatment outcomes. A previous systematic review in 2009 described the effect of HIV, DM, and alcohol misuse on MDRTB treatment outcomes, with the authors noting worse outcomes in patients with alcohol misuse, and no significant difference in outcomes in people with HIV or diabetes^3^. The data in this 2009 review, however, was quite limited, with only four studies reporting on the association between HIV- and DM-related MDRTB treatment outcomes^3^.

In contrast, this review reported on 34 studies with over 14,000 patients comparing outcomes by HIV status. We noted higher pooled relative risk of unsuccessful treatment outcome in PLWH. This appeared to be largely driven by an increase in mortality in PLWH. Further analysis suggested that the effect of HIV on mortality was increased in low income regions compared with high-income regions. Reasons for this remain unclear, as stratifying primary outcomes by study year, proportion with ART, and publication quality did not reveal any notable trends in study outcome. Given the preponderance of evidence demonstrating the mortality lowering effect of ART in co-infected patients^85,86^, we expected our results a trend towards improved outcomes in high ART settings. Surprisingly, however, there was no trend towards improved outcomes by study-level ART proportions. Further investigation into the drivers of mortality in the HIV-MDRTB co-infected populations is needed.

Those with alcohol misuse also had an increased pooled odds of unsuccessful treatment outcome. This appeared to be driven by default in people with alcohol misuse. Understanding the mechanisms behind the high default proportions will be critical in improving outcomes in MDR/XDRTB patients with a history of alcohol misuse. Interestingly, it seems that in the drug sensitive TB cohorts, alcohol misuse also predicts higher proportions of default^87,88^. This is thought to be primarily due to comorbid substance abuse, and socioeconomic conditions that prevent patients from accessing care reliably^87–90^. Programs have been developed to improve outcomes in people with alcohol use disorders in TB treatment and have been successful in decreasing loss to follow up in this population^46,91^.

We were somewhat surprised to find that people with diabetes had similar outcomes to people without diabetes in our analysis. Diabetes is associated with worse treatment outcomes in drug-susceptible TB, and is mentioned as a driver of poor TB treatment outcomes in several guidelines and reviews^7^. Unfortunately, due to lack of data, we were unable to explore the relationship between diabetes and individual MDR/XDRTB treatment outcomes. Similarly, the pooled primary outcome in smokers was not significantly different from non-smokers and we had insufficient data in secondary analysis to further investigate this relationship.

The strengths of this study include our broad search strategy, large sample size, and *a priori* study design. The studies included in this analysis reported treatment outcomes ranging over two decades and included studies from high, middle, and low-income regions. We also used clinically relevant variables consistent with accepted World Health Organization (WHO) treatment outcomes^1,4^.

There are also several notable weaknesses. First, we only estimated pooled effects on univariate analysis, and did not perform analysis that would control for potential confounding variables. Furthermore, in the vast majority of studies, there was not enough data available to assess potential confounding variables or perform meta-regression. In many comorbid conditions, confounding could potentially play a major role, as each of these comorbid conditions is associated with demographic, clinical, and socioeconomic characteristics that likely influence treatment outcomes. We explored some study-level variables and their influence on treatment outcomes, such as regional GDP, study quality, and publication year. These study-level variables, however, were of limited impact on pooled outcomes, and did not provide significant insight into the mechanisms through which comorbidities may impact treatment outcome. Ideally, an individual patient data meta-analysis (IPDMA) could be performed in an attempt to control for any confounding variables, however, this approach would likely limit sample size, and could potentially introduce bias, as better-resourced MDRTB/XDRTB treatment programs are more likely to compile, store and report such data for IPDMA.

Beyond confounding, other domains of bias may also be present and unaccounted for in studies included in our analysis. Specifically, some populations with co-morbidities may have had differential interventions (i.e. individuals with co-morbidities may receive more intensive treatment, monitoring, or support) which may bias outcomes in these populations. Additionally, we cannot exclude bias introduced from missing data, particularly given the relatively high proportions of default. Finally, bias introduced by selective reporting from better-resourced MDR-TB treatment programs may be present, as presumably well-resourced programs would be better equipped to treat individuals with MDR-TB and co-morbidities. We were unable to detect specific sources of bias when analyzing data by study quality, country income or ART coverage, but these sources of bias cannot be excluded.

The substantial between-study heterogeneity likely reflects a diversity of treatment conditions for MDR/XDRTB. Between-study heterogeneity was only partially reduced by analysis of study-level covariates (quality, country income category, and ART coverage). The presence of heterogeneity is not surprising, given the diversity of treatments, supports and approaches to MDR/XDRTB globally. Other likely sources of heterogeneity include programmatic factors such as treatment regimens, supports and infrastructure. Heterogeneity in study outcomes could also be attributed to the lack of standardized treatment outcomes in older studies. Unfortunately, we did not have the data to quantitatively assess each of these variables as potential sources of heterogeneity.

Recently, the World Health Organization (WHO) treatment guidelines for drug-resistant tuberculosis highlighted the need for “*inclusion and separate reporting of outcomes for key subgroups…especially children and HIV-positive individuals on treatment*”^92^. This systematic review highlights the need for improved reporting on a number of comorbid conditions in both MDRTB and XDRTB care. Other comorbidities not examined in this systematic review, such as mental illnesses and other substance use disorders, should also be considered for routine reporting in MDR/XDRTB treatment^93^. Improved reporting on outcomes related to specific comorbidities can help clarify the mechanisms that lead to unsuccessful treatment outcome for different subpopulations. This, in turn, would enable the development of new programs directed towards more individualized and appropriate MDRTB care and support.

## Electronic supplementary material

Supplemental Information
