# Supplementary material for: Comorbidities and treatment outcomes in multidrug resistant tuberculosis: a systematic review and meta-analysis
Source: Sci Rep. 2018 Mar 21;8:4980. doi: 10.1038/s41598-018-23344-z (PMC5862834; doi:10.1038/s41598-018-23344-z)
Supplement: Supplementary file 1 — Supplemental Information [file 41598_2018_23344_MOESM1_ESM.pdf]

Full Title: Comorbidities and treatment outcomes in multidrug resistant tuberculosis: a systematic review and meta-analysis

Short Title: Treatment outcomes in MDR/XDRTB

Authors and Credentials:

1) Joel Philip Samuels[1], BSc, MD (Corresponding Author)
    a. Email: j.samuels@alumni.ubc.ca
2) Aashna Sood[2]
3) Jonathon R. Campbell[3], BSc
4) Faiz Ahmad Khan[4,5,6], MD, MPH
5) James Cameron Johnston[7,8], MD, MPH (Alternate Corresponding Author)
    a. Email: James.Johnston@bccdc.ca

Affiliations:

1. Faculty of Medicine, University of British Columbia, Vancouver, British Columbia, Canada.
2. Royal College of Surgeons in Ireland, Dublin, Ireland.
3. Faculty of Pharmaceutical Sciences, University of British Columbia, Vancouver, British Columbia, Canada.
4. Respiratory Epidemiology & Clinical Research Unit, Centre for Outcomes Research and Evaluation, MUHC-RI, Montreal, Quebec, Canada.

5. Division of Respiratory Medicine, McGill University Health Centre, Montreal, Quebec, Canada.
6. McGill International TB Centre, Montreal, Quebec, Canada.
7. TB Services, BC Centre for Disease Control, Vancouver, British Columbia, Canada.
8. Division of Respiratory Medicine, University of British Columbia, Vancouver, British Columbia, Canada.

Registration: Protocol Registered in Prospero:
http://www.crd.york.ac.uk/PROSPERO/display_record.asp?ID=CRD42016039866

Conflicts of Interest: No authors have any relevant conflict of interests.

Supplemental Figure and Table Legend:

Supp. Appendix: Literature Review Search Strategy.

Supp. Fig. 1: Funnel plot for the effect of HIV on the primary outcome in MDR/XDRTB.

Supp. Fig. 2: Funnel plot for the effect of DM on the primary outcome in MDR/XDRTB.

Supp. Fig. 3: Funnel plot for the effect of smoking on the primary outcome in MDR/XDRTB.

Supp. Fig. 4: Funnel plot showing the effect of alcohol misuse on the primary outcome in MDR/XDRTB.

Supp. Fig. 5: Relative Risk of the primary outcome in MDR/XDRTB in those with and without DM in high GDP Countries.

Supp. Fig. 6: Relative Risk of the primary outcome in MDR/XDRTB in those with and without DM in low GDP Countries.

Supp. Fig. 7: Relative Risk of the primary outcome in MDR/XDRTB in those with and without DM from low to high quality study.

Supp. Fig. 8: Relative Risk of Default in MDR/XDRTB in those with HIV compared to those without HIV.

Supp. Fig. 9: Relative Risk of Treatment Failure in MDR/XDRTB among those with HIV compared to those without HIV.

Supp. Fig. 10: Relative Risk of Death/Failure in MDR/XDRTB in those with HIV compared those with and without HIV.

Supp. Fig. 11: Relative Risk of the primary outcome in MDR/XDRTB comparing those with and without HIV from low to high study quality.

Supp. Fig. 12: Relative Risk of the primary outcome in MDR/XDRTB comparing those with and without HIV in high GDP countries.

Supp. Fig. 13: Relative Risk of the primary outcome in MDR/XDRTB comparing those with and without HIV in low GDP countries.

Supp. Fig. 14: Relative Risk of the primary outcome in MDR/XDRTB comparing those with and without HIV from lowest to highest ART use.

Supp. Fig. 15: Relative Risk of the primary outcome in MDR/XDRTB comparing smokers and non-smokers from low to high quality study.

Supp. Fig. 16: Relative Risk of the primary outcome in MDR/XDRTB comparing smokers and non-smokers in high GDP countries.

Supp. Fig. 17: Relative Risk of the primary outcome in MDR/XDRTB comparing smokers and non-smokers in low GDP countries.

Supp. Fig. 18: Relative Risk of the primary outcome in MDR/XDRTB in those with alcohol misuse (AM) compared to those without alcohol misuse from low to high study quality.

Supp. Fig. 19: Relative Risk of the primary outcome in MDR/XDRTB in those with alcohol misuse (AM) compared to those without alcohol misuse in high GDP countries.

Supp. Fig. 20: Relative Risk of the primary outcome in MDR/XDRTB in those with alcohol misuse (AM) compared to those without alcohol misuse in low GDP countries.

Supp. Table 1: Proportion of data missing amongst participants with DM.

Supp. Table 2: Proportion of data missing amongst participants with HIV.

Supp. Table 3: Proportion of data missing amongst participants that smoke.

Supp. Table 4: Proportion of data missing amongst participants with alcohol misuse.

Supp. Table 5: Proportion of participants with HIV on ART.

Supp. Table 6: Definition of smoker used in study.

Supp. Table 7: Effect estimates for combined negative treatment outcomes in participants with DM from studies that did not provide raw data.

Supp. Table 8: Effect estimates for combined negative treatment outcomes in participants with HIV from studies that did not provide raw data.

Supp. Table 9: Effect estimates for combined negative treatment outcomes in participants that smoked from studies that did not provide raw data.

Supp. Table 10: Effect estimates combined negative treatment outcomes in participants with alcohol misuse from studies that did not provide raw data.

Appendix:

Search Strategy: We will conduct the following searches for each of the listed databases.

MEDLINE via OVID SP

1. Exp Tuberculosis, Multidrug-Resistant/
2. MDRTB.mp.
3. MDR TB.mp.
4. MDR-TB.mp.
5. MDR Tuberculosis.mp.
6. Multi*drug resistan* TB.mp.
7. Multi*drug resistan* Tuberculosis.mp.
8. (tuberculosis adj10 multi* drug resistan*).mp.
9. (tuberculosis adj10 MDR).mp.
10. (TB adj10 MDR).mp.
11. (TB adj10 multi* drug resistan*).mp.
12. extensive* drug resistan* tuberculosis.mp.
13. extensive* drug resistan* TB.mp.
14. XDR Tuberculosis.mp.
15. XDR TB.mp.
16. XDR-TB.mp.
17. XDRTB.mp.
18. (tuberculosis adj10 extensive* drug resistan*).mp.
19. (tuberculosis adj10 XDR).mp.
20. (TB adj10 XDR).mp.
21. (TB adj10 extensive* drug resistan*).mp.
22. exp Extensively Drug-Resistant Tuberculosis/
23. exp Treatment Outcome/
24. outcom*.mp.
25. 1 or 2 or 3 or 4 or 5 or 6 or 7 or 8 or 9 or 10 or 11 or 12 or 13 or 14 or 15 or 16 or 17 or 18 or 19 or 20 or 21 or 22
26. 23 or 24
27. 25 AND 26
28. limit 27 to English, French or Spanish language, Humans and 1980-2016

EMBASE via OVID SP

1. exp multidrug resistant Tuberculosis/
2. MDRTB.mp.
3. MDR TB.mp.
4. MDR-TB.mp.
5. Multi*drug resistan* TB.mp.
6. Multi*drug resistan* Tuberculosis.mp.
7. MDR Tuberculosis.mp.

8. (tuberculosis adj10 MDR).mp.
9. (tuberculosis adj10 multi* drug resistan*).mp.
10. (TB adj10 MDR).mp.
11. (TB adj10 multi* drug resistan*).mp.
12. exp extensively drug resistant tuberculosis/
13. extensive* drug resistan* TB.mp.
14. extensive* drug resistan* Tuberculosis.mp.
15. XDR Tuberculosis.mp.
16. XDRTB.mp.
17. XDR TB.mp.
18. XDR-TB.mp.
19. (tuberculosis adj10 extensive* drug resistan*).mp.
20. (tuberculosis adj10 XDR).mp.
21. (TB adj10 extensive* drug resistan*).mp.
22. (TB adj10 XDR).mp.
23. exp Treatment outcome/
24. outcom*.mp.
25. 1 or 2 or 3 or 4 or 5 or 6 or 7 or 8 or 9 or 10 or 11 or 12 or 13 or 14 or 15 or 16 or 17 or 18 or 19 or 20 or 21 or 22
26. 23 or 24
27. 25 AND 26
28. Limit 27 to English, French or Spanish language, Humans and 1980-2016

Cochrane Database of Systematic reviews via OVID

1. Multi*drug resistan* tuberculosis.mp.
2. Extensive* drug resistan* tuberculosis.mp.
3. XDR-TB.mp.
4. XDR TB.mp.
5. MDR TB.mp.
6. MDR-TB.mp.
7. (tuberculosis adj10 MDR).mp.
8. (tuberculosis adj10 multi* drug resistan*).mp.
9. (TB adj10 multi* drug resistan*).mp.
10. (TB adj10 MDR).mp.
11. (tuberculosis adj10 XDR).mp.
12. (tuberculosis adj10 extensive* drug resistan*).mp.
13. (TB adj10 XDR).mp.
14. (TB adj10 extensive* drug resistan*).mp.
15. 1 or 2 or 3 or 4 or 5 or 6 or 7 or 8 or 9 or 10 or 11 or 12 or 13 or 14

Cochrane Central Register of Controlled Trials via OVID

1. exp Tuberculosis, Multidrug-Resistant/
2. exp Extensively Drug-Resistant Tuberculosis/
3. extensive* drug resistan* Tuberculosis.mp.

4. extensive* drug resistan* TB.mp.
5. XDRTB.mp.
6. XDR-TB.mp.
7. XDR TB.mp.
8. XDR Tuberculosis.mp.
9. MDRTB.mp.
10. MDR-TB.mp.
11. MDR TB.mp.
12. MDR Tuberculosis.mp.
13. Multi*drug resistan* Tuberculosis.mp.
14. Multi*drug resistan* TB.mp.
15. (tuberculosis adj10 extensive* drug resistan*).mp.
16. (tuberculosis adj10 XDR).mp.
17. (tuberculosis adj10 MDR).mp.
18. (tuberculosis adj10 multi* drug resistan*).mp.
19. (TB adj10 XDR).mp.
20. (TB adj10 extensive* drug resistan*).mp.
21. (TB adj10 multi* drug resistan*).mp.
22. (TB adj10 MDR).mp.
23. 1 or 2 or 3 or 4 or 5 or 6 or 7 or 8 or 9 or 10 or 11 or 12 or 13 or 14 or 15 or 16 or 17 or 18 or 19 or 20 or 21 or 22
24. Limit 19 to English, French or Spanish Languages and 1980-2016

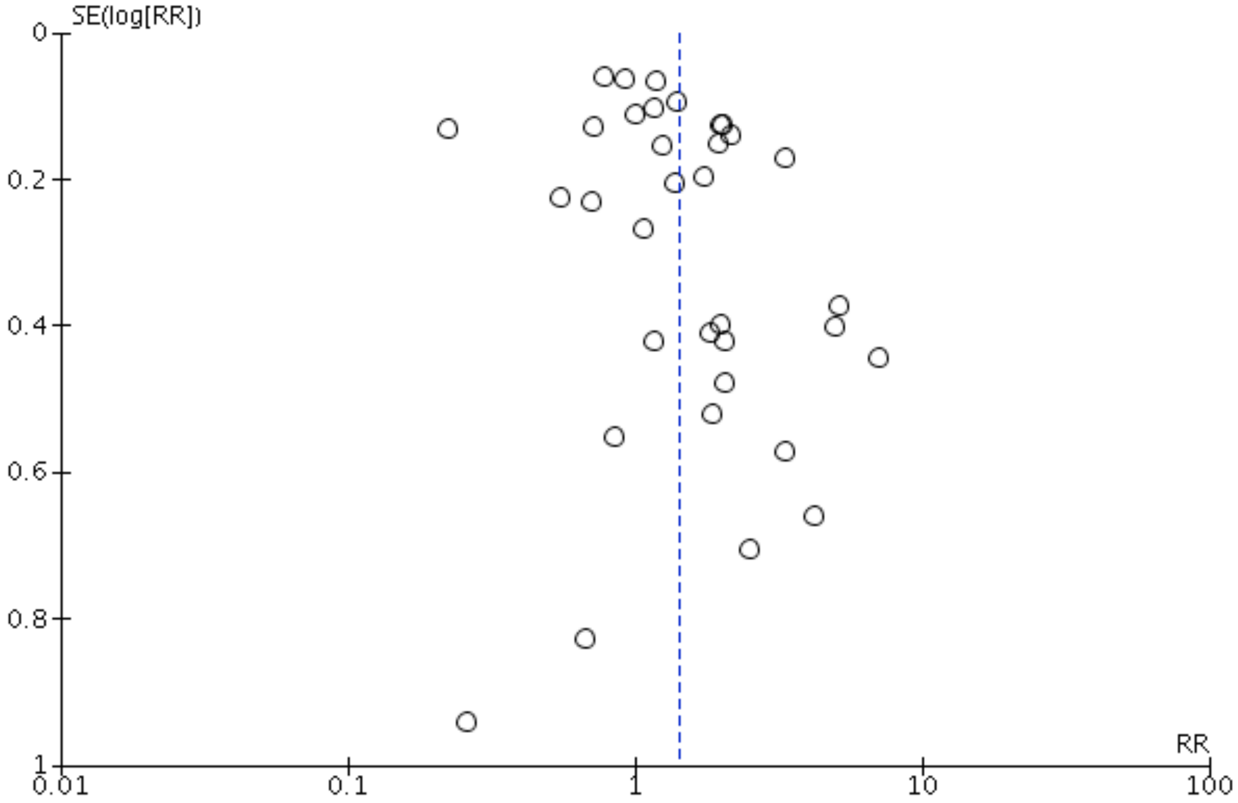

Supp. Fig. 1: Funnel plot for the effect of HIV on the primary outcome in MDR/XDRTB.

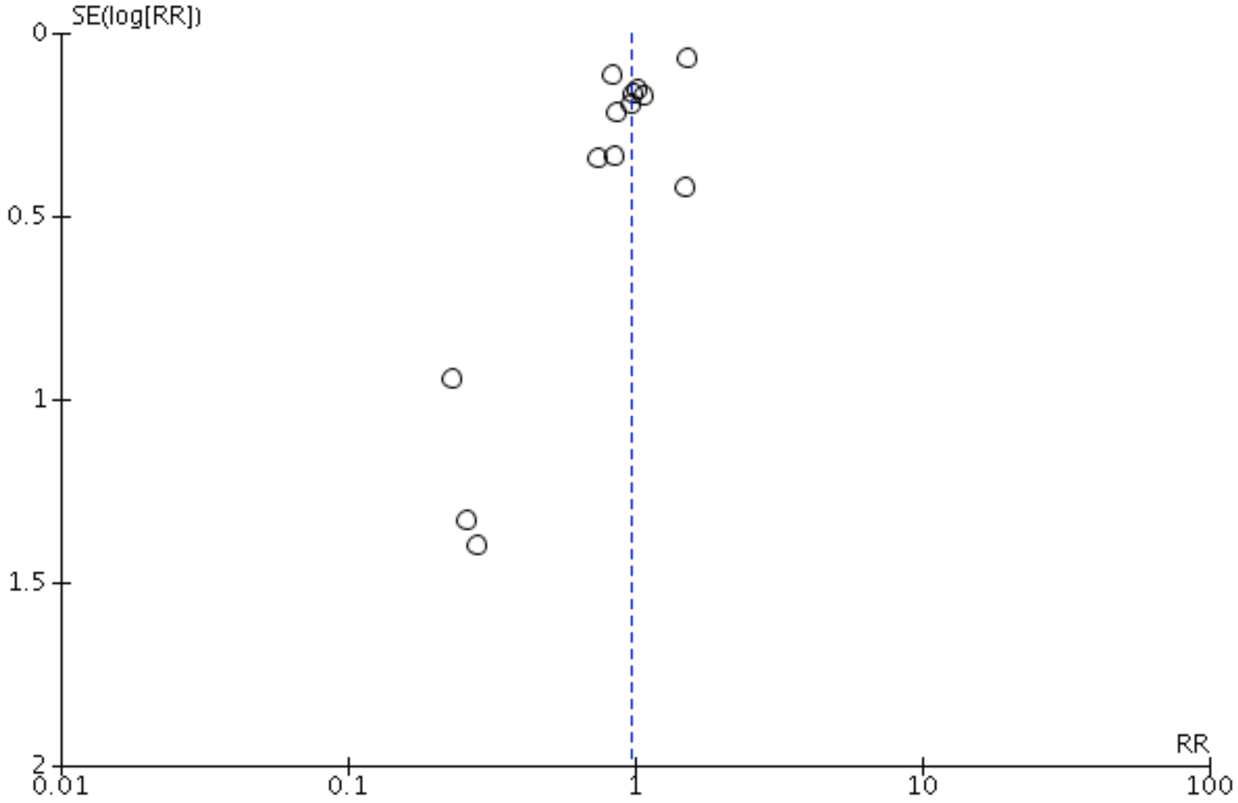

Supp. Fig. 2: Funnel plot for the effect of DM on the primary outcome in MDR/XDRTB.

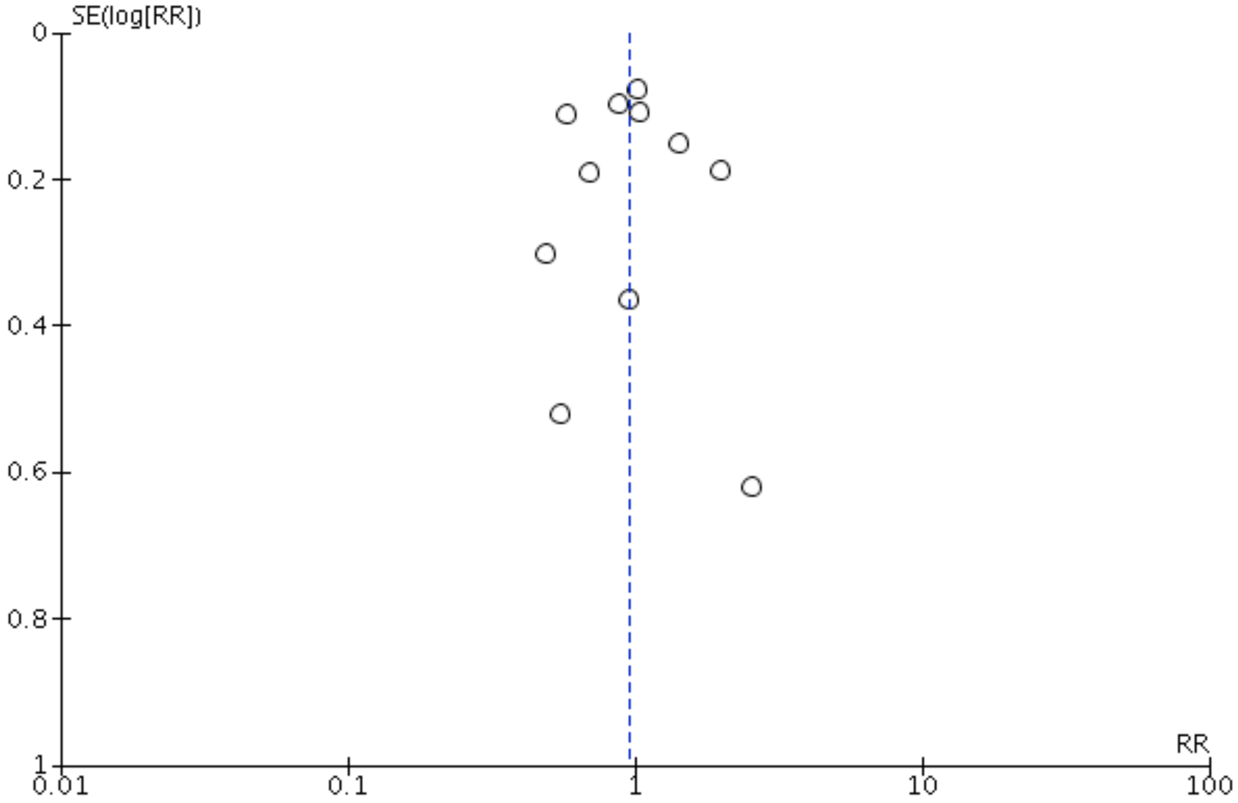

Supp. Fig. 3: Funnel plot for the effect of smoking on the primary outcome in MDR/XDRTB.

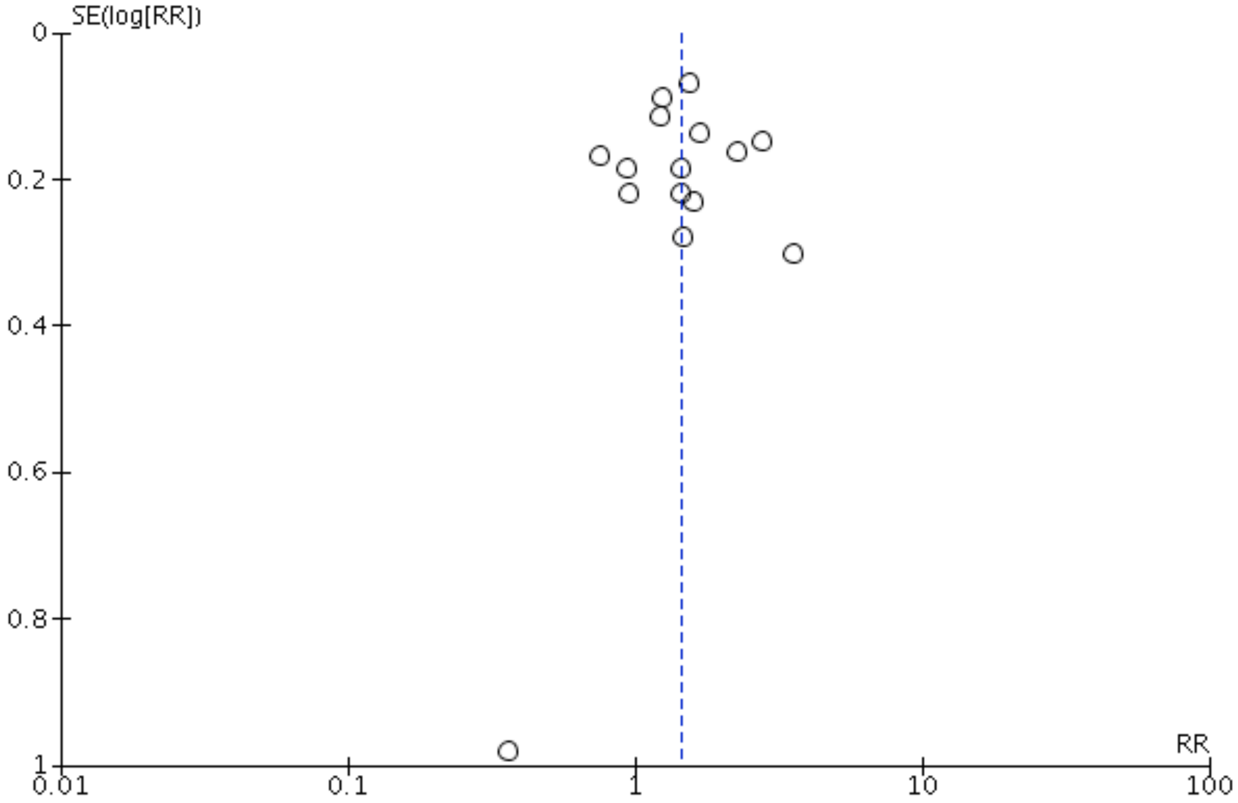

Supp. Fig. 4: Funnel plot showing the effect of alcohol misuse on the primary outcome in MDR/XDRTB.

Study or Subgroup	Diabetics Events	Diabetics Total	Non-Diabetics Events	Non-Diabetics Total	Weight	Risk Ratio M-H, Random, 95% CI
Bendayan et al. 2011	6	17	56	118	11.9%	0.74 [0.38, 1.45]
Jeon et al. 2009	17	26	97	150	20.8%	1.01 [0.75, 1.37]
Kuksa et al. 2014	0	3	63	130	1.4%	0.26 [0.02, 3.47]
Kwak et al. 2015	0	10	18	133	1.3%	0.33 [0.02, 5.10]
Leimane et al. 2005	3	6	66	198	9.3%	1.50 [0.66, 3.42]
Gegia et al 2012	16	35	163	345	18.8%	0.97 [0.66, 1.41]
Kempker et al. 2015	6	16	55	125	12.0%	0.85 [0.44, 1.65]
Tang et al. 2013	65	80	269	506	24.6%	1.53 [1.34, 1.75]
**Total (95% CI)**		**193**		**1705**	**100.0%**	**1.05 [0.77, 1.44]**
Total events	113		787			

Heterogeneity: $Tau^2 = 0.10$; $Chi^2 = 21.92$, df = 7 (P = 0.003); $I^2 = 68\%$
Test for overall effect: Z = 0.32 (P = 0.75)

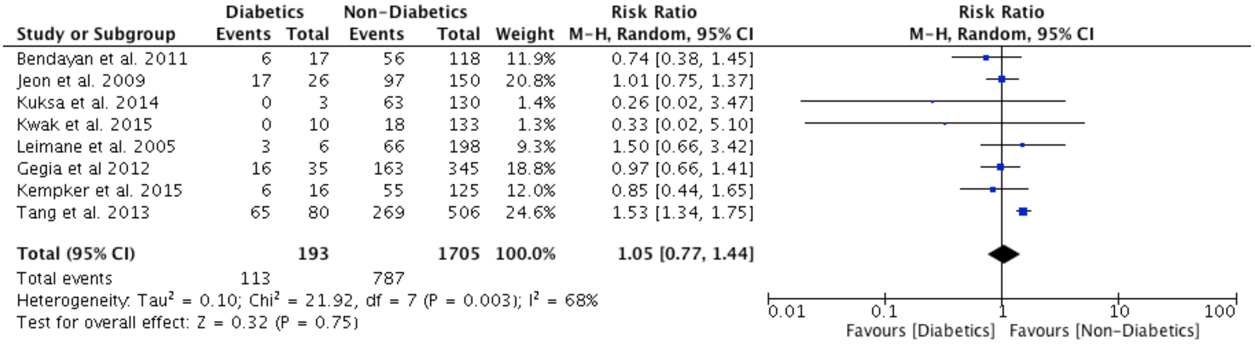

Supp. Fig. 5: Relative Risk of the primary outcome in MDR/XDRTB in those with and without DM in high GDP Countries.

Study or Subgroup	Diabetics Events	Diabetics Total	Non-Diabetics Events	Non-Diabetics Total	Weight	Risk Ratio M-H, Random, 95% CI
Akshata et al. 2016	1	8	33	61	2.8%	0.23 [0.04, 1.47]
Duraisamy et al. 2014	20	60	46	119	40.4%	0.86 [0.56, 1.32]
Gler et al. 2013	34	117	95	322	56.8%	0.98 [0.71, 1.37]
**Total (95% CI)**		**185**		**502**	**100.0%**	**0.90 [0.65, 1.23]**
Total events	55		174			

Heterogeneity: $Tau^2 = 0.02$; $Chi^2 = 2.48$, df = 2 (P = 0.29); $I^2 = 19\%$

Test for overall effect: Z = 0.69 (P = 0.49)

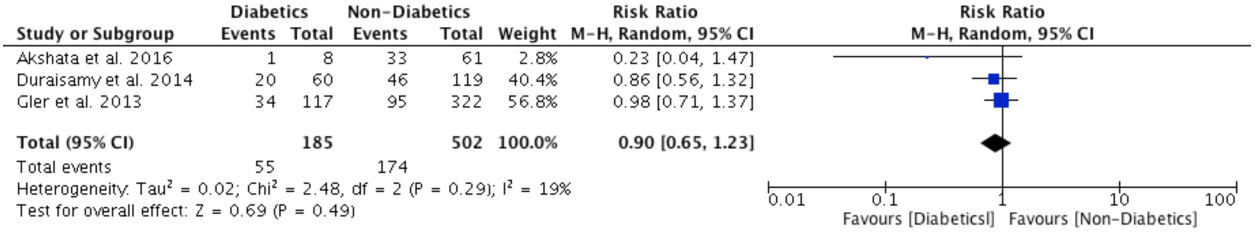

Supp. Fig. 6: Relative Risk of the primary outcome in MDR/XDRTB in those with and without DM in low GDP Countries.

	Diabetics		Non-Diabetics			Risk Ratio
Study or Subgroup	Events	Total	Events	Total	Weight	M-H, Random, 95% CI
Akshata et al. 2016	1	8	33	61	0.4%	0.23 [0.04, 1.47]
Kempker et al. 2015	6	16	55	125	3.4%	0.85 [0.44, 1.65]
Kurbatova et al. 2012	23	68	536	1700	12.9%	1.07 [0.76, 1.51]
Leimane et al. 2005	3	6	66	198	2.2%	1.50 [0.66, 3.42]
Bendayan et al. 2011	6	17	56	118	3.3%	0.74 [0.38, 1.45]
Duraisamy et al. 2014	20	60	46	119	8.3%	0.86 [0.56, 1.32]
Gegia et al 2012	16	35	163	345	10.4%	0.97 [0.66, 1.41]
Jeon et al. 2009	17	26	97	150	16.1%	1.01 [0.75, 1.37]
Kwak et al. 2015	0	10	18	113	0.2%	0.28 [0.02, 4.34]
Gler et al. 2013	34	117	95	322	13.7%	0.98 [0.71, 1.37]
Cegielski et al. 2016	55	162	428	1043	28.9%	0.83 [0.66, 1.04]
Kuksa et al. 2014	0	3	63	130	0.2%	0.26 [0.02, 3.47]
**Total (95% CI)**		**528**		**4424**	**100.0%**	**0.92 [0.82, 1.04]**
Total events	181		1656			

Heterogeneity: Tau² = 0.00; Chi² = 8.21, df = 11 (P = 0.69); I² = 0%
Test for overall effect: Z = 1.28 (P = 0.20)

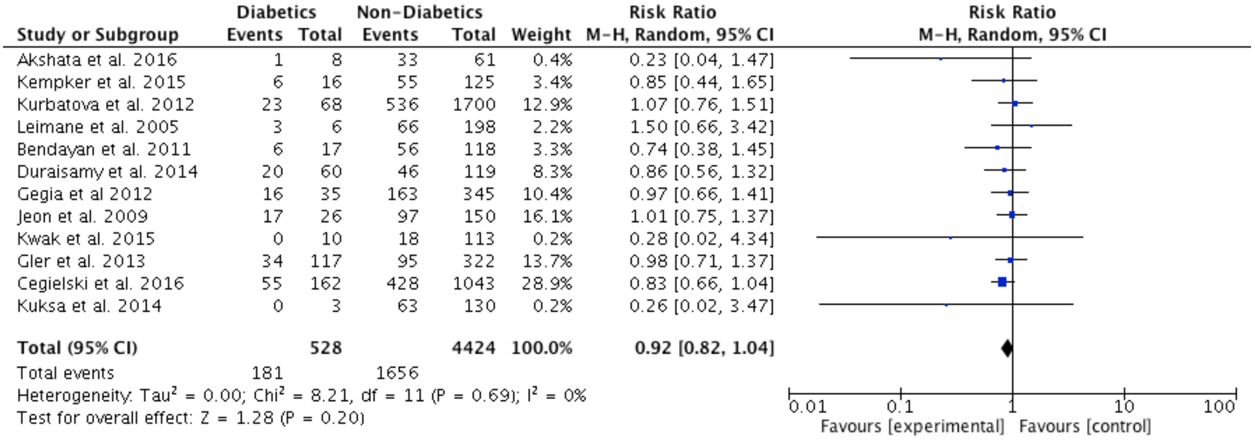

Supp. Fig. 7: Relative Risk of the primary outcome in MDR/XDRTB in those with and without DM from low to high quality study.

Study or Subgroup	HIV Events	HIV Total	Non-HIV Events	Non-HIV Total	Weight	Risk Ratio M-H, Random, 95% CI	Year
Brust et al. 2010	81	362	171	847	25.7%	1.11 [0.88, 1.40]	2010
Farley et al. 2011	59	287	99	470	23.0%	0.98 [0.73, 1.30]	2011
Satti et al. 2012	0	94	1	40	0.6%	0.14 [0.01, 3.46]	2012
Kurbatova et al. 2012	8	27	233	1741	11.3%	2.21 [1.22, 4.01]	2012
Charles et al. 2014	1	27	3	83	1.2%	1.02 [0.11, 9.45]	2014
van Altena et al. 2015	2	14	6	99	2.5%	2.36 [0.53, 10.56]	2015
Loveday et al. 2015	225	1104	112	445	27.5%	0.81 [0.66, 0.99]	2015
Van Der Walt et al. 2016	15	393	12	278	8.2%	0.88 [0.42, 1.86]	2016
**Total (95% CI)**		**2308**		**4003**	**100.0%**	**1.05 [0.82, 1.35]**	
Total events	391		637				

Heterogeneity: $Tau^2 = 0.05$; $Chi^2 = 14.70$, df = 7 (P = 0.04); $I^2 = 52\%$

Test for overall effect: Z = 0.42 (P = 0.67)

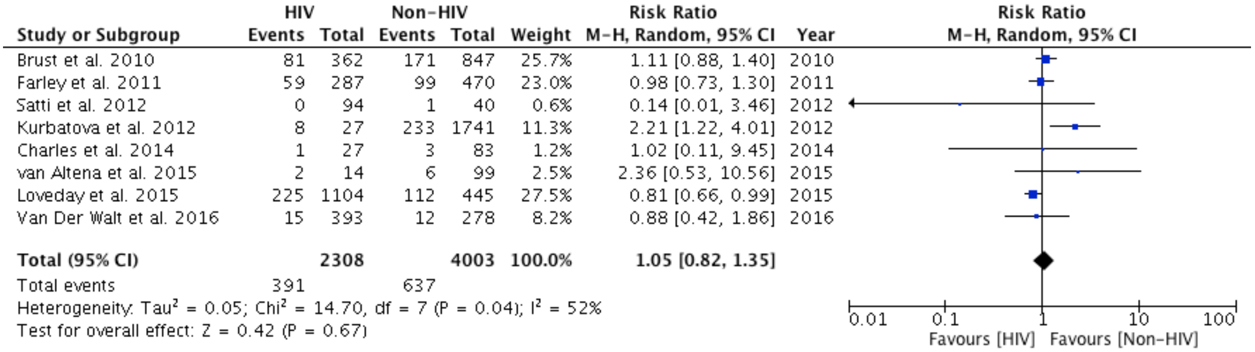

Supp. Fig. 8: Relative Risk of Default in MDR/XDRTB in those with HIV compared to those without HIV.

Study or Subgroup	HIV Events	HIV Total	Non-HIV Events	Non-HIV Total	Weight	Risk Ratio M-H, Random, 95% CI
Farley et al. 2011	12	287	62	470	22.8%	0.32 [0.17, 0.58]
Khaliaukin et al. 2014	8	67	72	372	21.1%	0.62 [0.31, 1.22]
Kurbatova et al. 2012	1	27	117	1741	6.3%	0.55 [0.08, 3.80]
Loveday et al. 2015	55	1104	23	445	25.5%	0.96 [0.60, 1.55]
Meressa et al. 2015	4	133	6	479	11.8%	2.40 [0.69, 8.38]
Satti et al. 2012	1	94	0	40	2.6%	1.29 [0.05, 31.12]
Van Der Walt et al. 2016	5	393	3	278	9.9%	1.18 [0.28, 4.89]
**Total (95% CI)**		**2105**		**3825**	**100.0%**	**0.75 [0.44, 1.29]**
Total events	86		283			

Heterogeneity: $Tau^2 = 0.24$; $Chi^2 = 13.21$, df = 6 (P = 0.04); $I^2 = 55\%$

Test for overall effect: Z = 1.03 (P = 0.30)

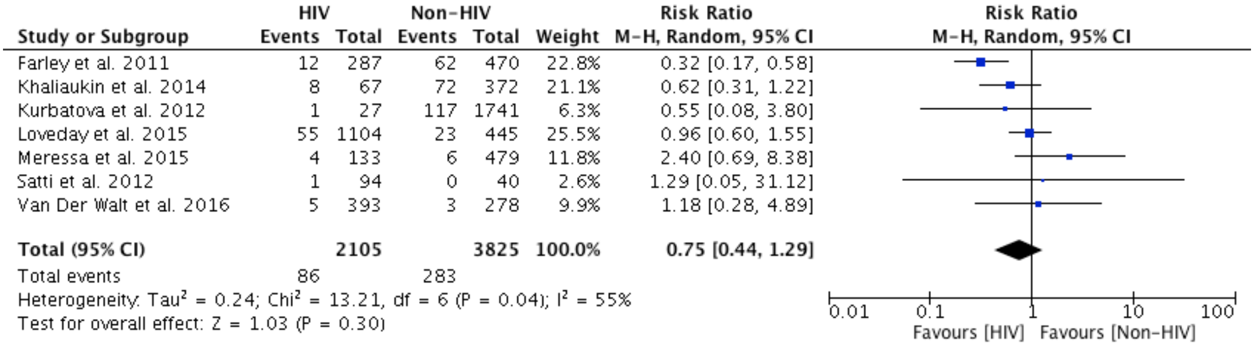

Supp. Fig. 9: Relative Risk of Treatment Failure in MDR/XDRTB among those with HIV compared to those without HIV.

Study or Subgroup	HIV Events	HIV Total	Non-HIV Events	Non-HIV Total	Weight	Risk Ratio M-H, Random, 95% CI	Year
Park et al. 1996	68	90	32	83	4.6%	1.96 [1.46, 2.63]	1996
Shah et al. 2008	21	31	5	52	2.6%	7.05 [2.96, 16.78]	2008
O'Donnell et al. 2009	18	43	7	17	3.2%	1.02 [0.52, 1.98]	2009
Brust et al. 2010	90	362	341	847	4.9%	0.62 [0.51, 0.75]	2010
Dheda et al. 2010	34	82	28	92	4.2%	1.36 [0.91, 2.04]	2010
Bendayan et al. 2011	1	8	52	127	0.9%	0.31 [0.05, 1.93]	2011
Farley et al. 2011	113	287	138	470	4.9%	1.34 [1.10, 1.64]	2011
Kvasnovsky et al. 2011	55	108	40	98	4.6%	1.25 [0.92, 1.69]	2011
Seddon et al. 2012	7	40	6	68	2.2%	1.98 [0.72, 5.49]	2012
Gegia et al 2012	2	5	94	375	2.0%	1.60 [0.54, 4.74]	2012
Satti et al. 2012	30	94	17	40	4.0%	0.75 [0.47, 1.20]	2012
Gandhi et al. 2012	149	200	40	62	4.9%	1.15 [0.94, 1.41]	2012
Kurbatova et al. 2012	10	27	308	1741	3.8%	2.09 [1.27, 3.46]	2012
Oliveira et al. 2013	63	108	50	256	4.6%	2.99 [2.22, 4.01]	2013
Duraisamy et al. 2014	1	1	38	178	2.6%	3.49 [1.49, 8.14]	2014
Kuksa et al. 2014	8	16	39	117	3.6%	1.50 [0.86, 2.61]	2014
Marais et al. 2014	45	203	28	121	4.2%	0.96 [0.63, 1.45]	2014
Charles et al. 2014	5	27	6	83	2.0%	2.56 [0.85, 7.73]	2014
Chung-Delgado et al. 2014	4	10	42	191	2.8%	1.82 [0.81, 4.07]	2014
Khaliaukin et al. 2014	47	67	187	372	4.9%	1.40 [1.16, 1.68]	2014
Oladimeji et al. 2014	7	28	17	134	2.8%	1.97 [0.90, 4.30]	2014
van Altena et al. 2015	5	14	4	99	1.8%	8.84 [2.69, 29.05]	2015
Meressa et al. 2015	31	133	64	479	4.3%	1.74 [1.19, 2.56]	2015
Loveday et al. 2015	235	1104	89	445	4.8%	1.06 [0.86, 1.32]	2015
Van Der Walt et al. 2016	158	393	49	278	4.6%	2.28 [1.72, 3.02]	2016
Akshata et al. 2016	1	3	23	66	1.1%	0.96 [0.19, 4.90]	2016
Phuong et al. 2016	23	53	173	1327	4.5%	3.33 [2.38, 4.66]	2016
Cegielski et al. 2016	46	159	205	1085	4.7%	1.53 [1.17, 2.01]	2016
**Total (95% CI)**		**3696**		**9303**	**100.0%**	**1.61 [1.32, 1.96]**	
Total events	1277		2122				

Heterogeneity: $Tau^2 = 0.20$; $Chi^2 = 192.67$, df = 27 ($P < 0.00001$); $I^2 = 86\%$
Test for overall effect: Z = 4.74 ($P < 0.00001$)

Risk Ratio
M-H, Random, 95% CI

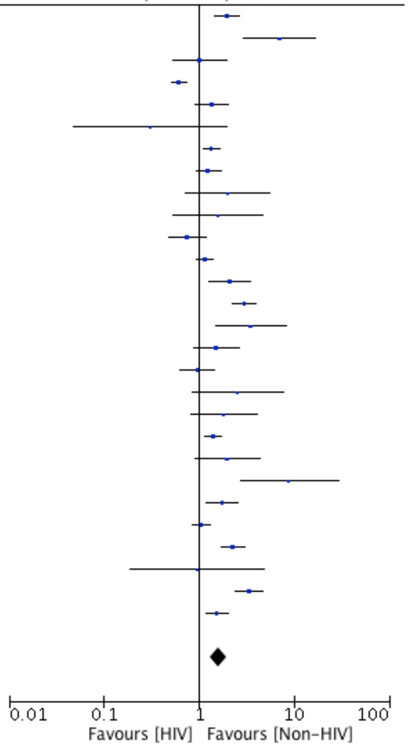

Supp. Fig. 10: Relative Risk of Death/Failure in MDR/XDRTB in those with HIV compared those with and without HIV.

Study or Subgroup	HIV Events	HIV Total	Non-HIV Events	Non-HIV Total	Weight	Risk Ratio M-H, Random, 95% CI
Akshata et al. 2016	1	3	33	66	1.1%	0.67 [0.13, 3.36]
Kempker et al. 2015	3	6	58	135	2.4%	1.16 [0.51, 2.65]
Kurbatova et al. 2012	18	27	541	1741	3.7%	2.15 [1.63, 2.83]
Gandhi et al. 2012	149	200	40	62	3.9%	1.15 [0.94, 1.41]
Kuaban et al. 2015	5	30	6	120	1.8%	3.33 [1.09, 10.19]
Oladimeji et al. 2014	7	28	17	134	2.5%	1.97 [0.90, 4.30]
Bendayan et al. 2011	1	8	61	127	0.9%	0.26 [0.04, 1.64]
Brust et al. 2010	171	362	512	847	3.9%	0.78 [0.69, 0.88]
Charles et al. 2014	6	27	9	83	2.2%	2.05 [0.80, 5.23]
Chung-Delgado et al. 2014	4	10	42	191	2.5%	1.82 [0.81, 4.07]
Duraisamy et al. 2014	1	1	65	178	2.4%	2.05 [0.90, 4.67]
Gegia et al 2012	2	5	177	375	1.9%	0.85 [0.29, 2.49]
Hicks et al. 2014	16	64	2	20	1.4%	2.50 [0.63, 9.95]
Kvasnovsky et al. 2011	55	108	40	98	3.7%	1.25 [0.92, 1.69]
Marais et al. 2014	45	203	121	121	3.8%	0.22 [0.17, 0.29]
O'Donnell et al. 2009	18	43	13	17	3.4%	0.55 [0.35, 0.85]
Park et al. 1996	68	90	32	83	3.7%	1.96 [1.46, 2.63]
Eker et al. 2008	2	7	12	177	1.5%	4.21 [1.16, 15.35]
Meressa et al. 2015	31	133	64	479	3.5%	1.74 [1.19, 2.56]
Van Der Walt et al. 2016	173	393	61	278	3.8%	2.01 [1.57, 2.57]
Cegielski et al. 2016	46	159	437	1085	3.8%	0.72 [0.56, 0.93]
Dheda et al. 2010	34	82	28	92	3.5%	1.36 [0.91, 2.04]
Dolgusev et al. 2014	11	39	12	234	2.6%	5.50 [2.61, 11.58]
Elliott et al. 2014	53	93	102	179	3.8%	1.00 [0.80, 1.24]
Farley et al. 2011	172	287	237	470	3.9%	1.19 [1.04, 1.35]
Khaliaukin et al. 2014	47	67	187	372	3.9%	1.40 [1.16, 1.68]
Kuksa et al. 2014	8	16	55	117	3.2%	1.06 [0.63, 1.80]
Loveday et al. 2015	460	1104	201	445	3.9%	0.92 [0.81, 1.04]
Oliveira et al. 2013	63	108	75	256	3.8%	1.99 [1.55, 2.55]
Phuong et al. 2016	23	53	173	1327	3.6%	3.33 [2.38, 4.66]
Satti et al. 2012	30	94	18	40	3.3%	0.71 [0.45, 1.11]
Seddon et al. 2012	7	43	6	68	2.0%	1.84 [0.66, 5.12]
Shah et al. 2008	21	31	5	52	2.3%	7.05 [2.96, 16.78]
van Altena et al. 2015	7	14	10	99	2.5%	4.95 [2.25, 10.88]
**Total (95% CI)**		**3938**		**10168**	**100.0%**	**1.41 [1.16, 1.73]**
Total events	1758		3452			

Heterogeneity: $Tau^2 = 0.27$; $Chi^2 = 420.59$, df = 33 ($P < 0.00001$); $I^2 = 92\%$

Test for overall effect: Z = 3.36 (P = 0.0008)

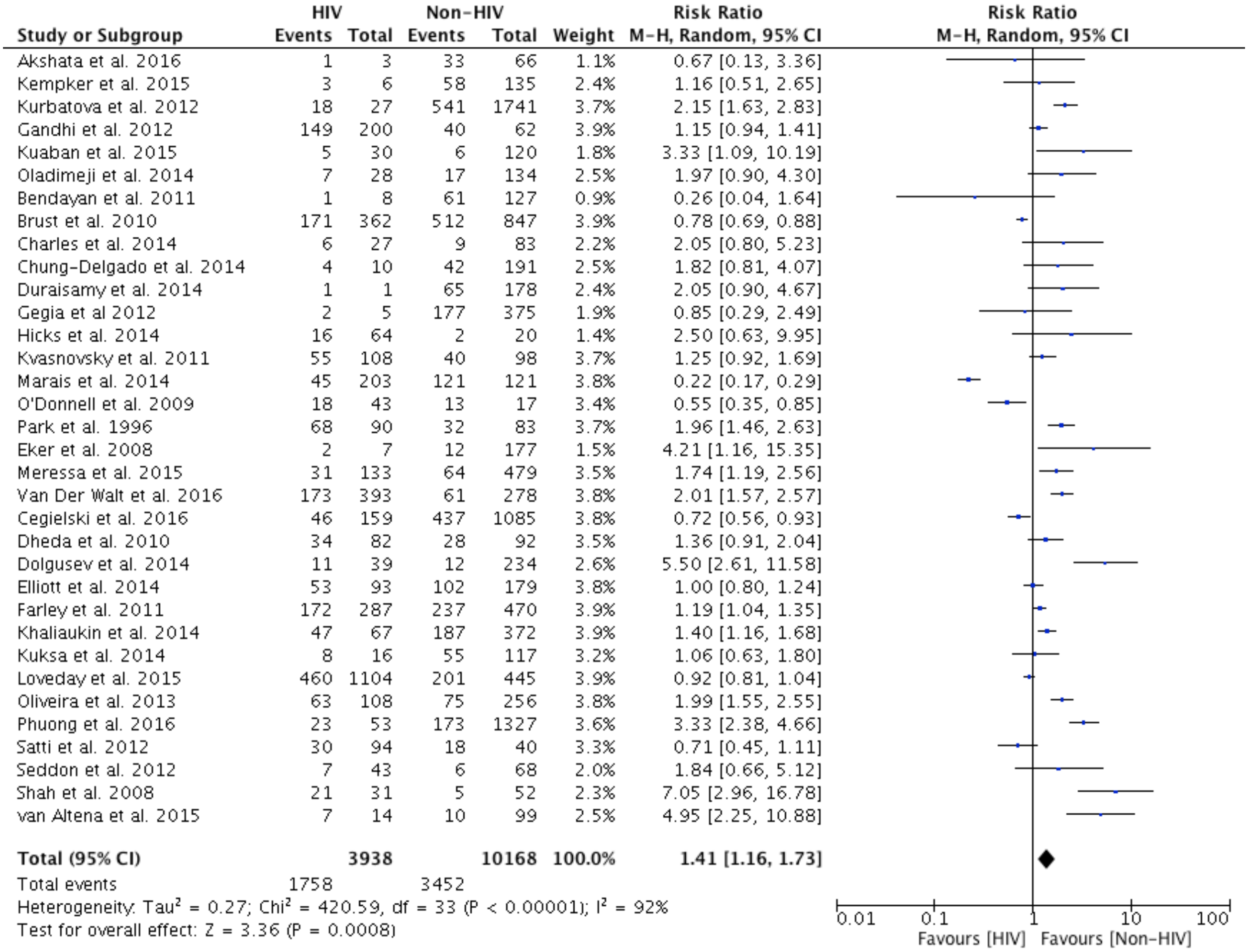

Supp. Fig. 11: Relative Risk of the primary outcome in MDR/XDRTB comparing those with and without HIV from low to high study quality.

Study or Subgroup	HIV Events	HIV Total	Non-HIV Events	Non-HIV Total	Weight	Risk Ratio M-H, Random, 95% CI
Bendayan et al. 2011	1	8	61	127	1.2%	0.26 [0.04, 1.64]
Brust et al. 2010	171	362	512	847	5.7%	0.78 [0.69, 0.88]
Dheda et al. 2010	34	82	28	92	4.9%	1.36 [0.91, 2.04]
Eker et al. 2008	2	7	12	177	2.0%	4.21 [1.16, 15.35]
Elliott et al. 2014	53	93	102	179	5.5%	1.00 [0.80, 1.24]
Farley et al. 2011	172	287	237	470	5.7%	1.19 [1.04, 1.35]
Gandhi et al. 2012	149	200	40	62	5.6%	1.15 [0.94, 1.41]
Gegia et al 2012	2	5	177	375	2.5%	0.85 [0.29, 2.49]
Hicks et al. 2014	16	64	2	20	1.8%	2.50 [0.63, 9.95]
Kempker et al. 2015	3	6	58	135	3.3%	1.16 [0.51, 2.65]
Khaliaukin et al. 2014	47	67	187	372	5.6%	1.40 [1.16, 1.68]
Kuksa et al. 2014	8	16	55	117	4.4%	1.06 [0.63, 1.80]
Kvasnovsky et al. 2011	55	108	40	98	5.3%	1.25 [0.92, 1.69]
Loveday et al. 2015	460	1104	201	445	5.7%	0.92 [0.81, 1.04]
Marais et al. 2014	45	203	121	121	5.4%	0.22 [0.17, 0.29]
O'Donnell et al. 2009	18	43	13	17	4.8%	0.55 [0.35, 0.85]
Oliveira et al. 2013	63	108	75	256	5.5%	1.99 [1.55, 2.55]
Park et al. 1996	68	90	32	83	5.3%	1.96 [1.46, 2.63]
Satti et al. 2012	30	94	18	40	4.7%	0.71 [0.45, 1.11]
Seddon et al. 2012	7	43	6	68	2.7%	1.84 [0.66, 5.12]
Shah et al. 2008	21	31	5	52	3.2%	7.05 [2.96, 16.78]
van Altena et al. 2015	7	14	10	99	3.4%	4.95 [2.25, 10.88]
Van Der Walt et al. 2016	173	393	61	278	5.5%	2.01 [1.57, 2.57]
**Total (95% CI)**		**3428**		**4530**	**100.0%**	**1.22 [0.97, 1.53]**
Total events	1605		2053			

Heterogeneity: $Tau^2 = 0.23$; $Chi^2 = 305.71$, df = 22 ($P < 0.00001$); $I^2 = 93\%$
Test for overall effect: Z = 1.69 (P = 0.09)

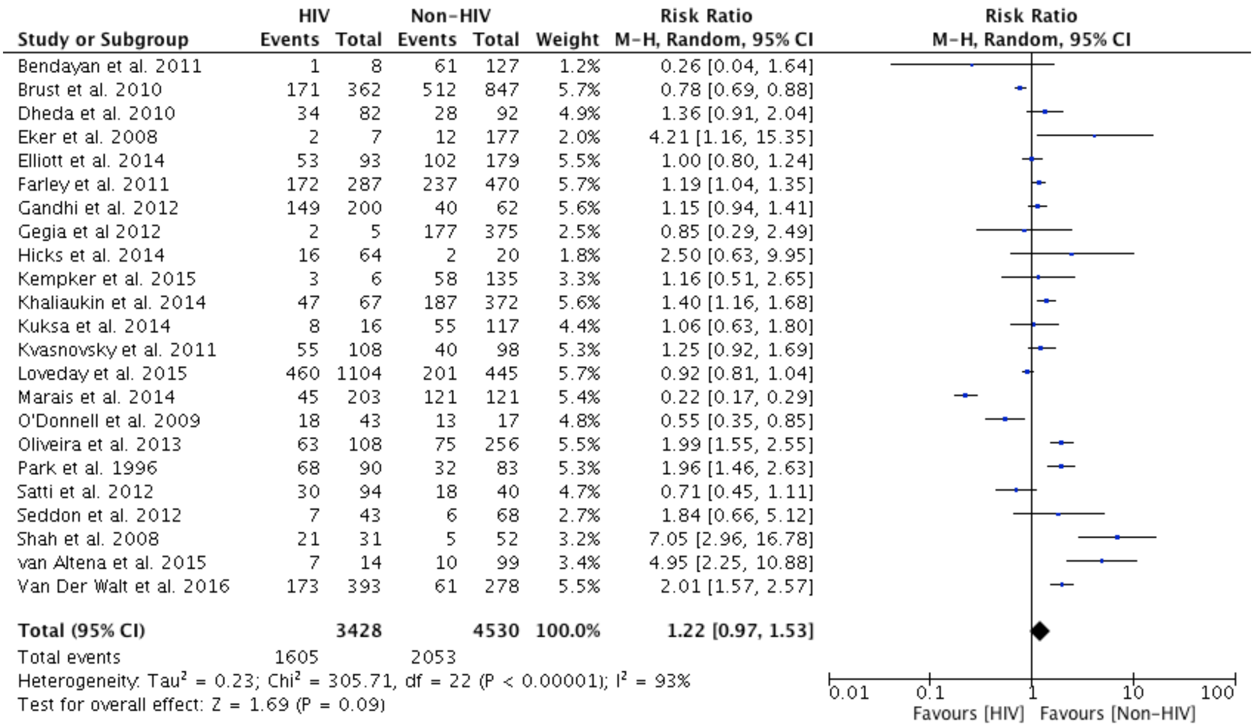

Supp. Fig. 12: Relative Risk of the primary outcome in MDR/XDRTB comparing those with and without HIV in high GDP countries.

Study or Subgroup	HIV Events	HIV Total	Non-HIV Events	Non-HIV Total	Weight	Risk Ratio M-H, Random, 95% CI
Akshata et al. 2016	1	3	33	66	3.9%	0.67 [0.13, 3.36]
Charles et al. 2014	6	27	9	83	9.7%	2.05 [0.80, 5.23]
Duraisamy et al. 2014	1	1	65	178	11.7%	2.05 [0.90, 4.67]
Kuaban et al. 2015	5	30	6	120	7.3%	3.33 [1.09, 10.19]
Meressa et al. 2015	31	133	64	479	26.3%	1.74 [1.19, 2.56]
Oladimeji et al. 2014	7	28	17	134	12.6%	1.97 [0.90, 4.30]
Phuong et al. 2016	23	53	173	1327	28.6%	3.33 [2.38, 4.66]
**Total (95% CI)**		**275**		**2387**	**100.0%**	**2.23 [1.60, 3.11]**
Total events	74		367			

Heterogeneity: $Tau^2 = 0.07$; $Chi^2 = 10.10$, df = 6 (P = 0.12); $I^2 = 41\%$

Test for overall effect: Z = 4.70 (P < 0.00001)

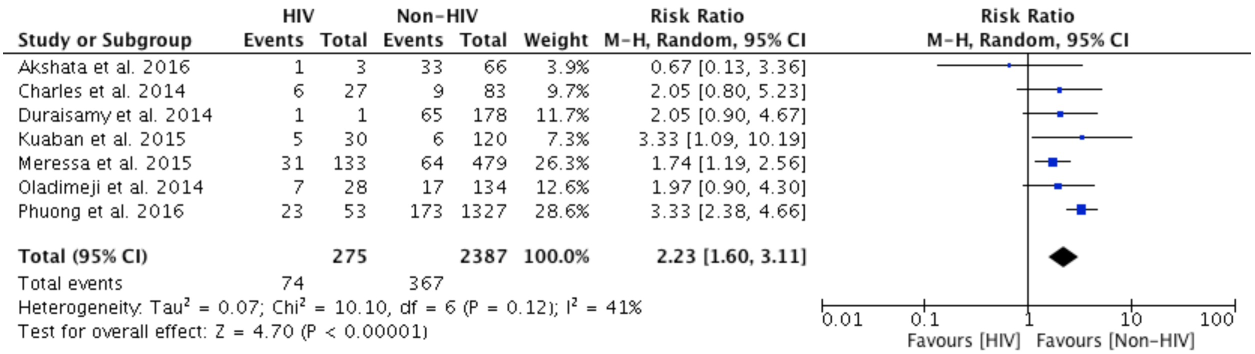

Supp. Fig. 13: Relative Risk of the primary outcome in MDR/XDRTB comparing those with and without HIV in low GDP countries.

Study or Subgroup	HIV Events	HIV Total	Non-HIV Events	Non-HIV Total	Weight	Risk Ratio M-H, Random, 95% CI
Van Der Walt et al. 2016	173	393	61	278	9.6%	2.01 [1.57, 2.57]
Gandhi et al. 2012	149	200	40	62	9.9%	1.15 [0.94, 1.41]
Khaliaukin et al. 2014	47	67	187	372	10.0%	1.40 [1.16, 1.68]
Phuong et al. 2016	23	53	173	1327	8.9%	3.33 [2.38, 4.66]
Kvasnovsky et al. 2011	55	108	40	98	9.2%	1.25 [0.92, 1.69]
Dheda et al. 2010	34	82	28	92	8.3%	1.36 [0.91, 2.04]
O'Donnell et al. 2009	18	43	13	17	8.0%	0.55 [0.35, 0.85]
Kuaban et al. 2015	5	30	6	120	3.4%	3.33 [1.09, 10.19]
Loveday et al. 2015	460	1104	201	445	10.3%	0.92 [0.81, 1.04]
Satti et al. 2012	30	94	18	40	7.9%	0.71 [0.45, 1.11]
Meressa et al. 2015	31	133	64	479	8.5%	1.74 [1.19, 2.56]
Akshata et al. 2016	1	3	33	66	2.0%	0.67 [0.13, 3.36]
Charles et al. 2014	6	27	9	83	4.3%	2.05 [0.80, 5.23]
**Total (95% CI)**		**2337**		**3479**	**100.0%**	**1.34 [1.04, 1.72]**
Total events	1032		873			

Heterogeneity: $Tau^2 = 0.15$; $Chi^2 = 98.12$, $df = 12$ ($P < 0.00001$); $I^2 = 88\%$

Test for overall effect: $Z = 2.30$ ($P = 0.02$)

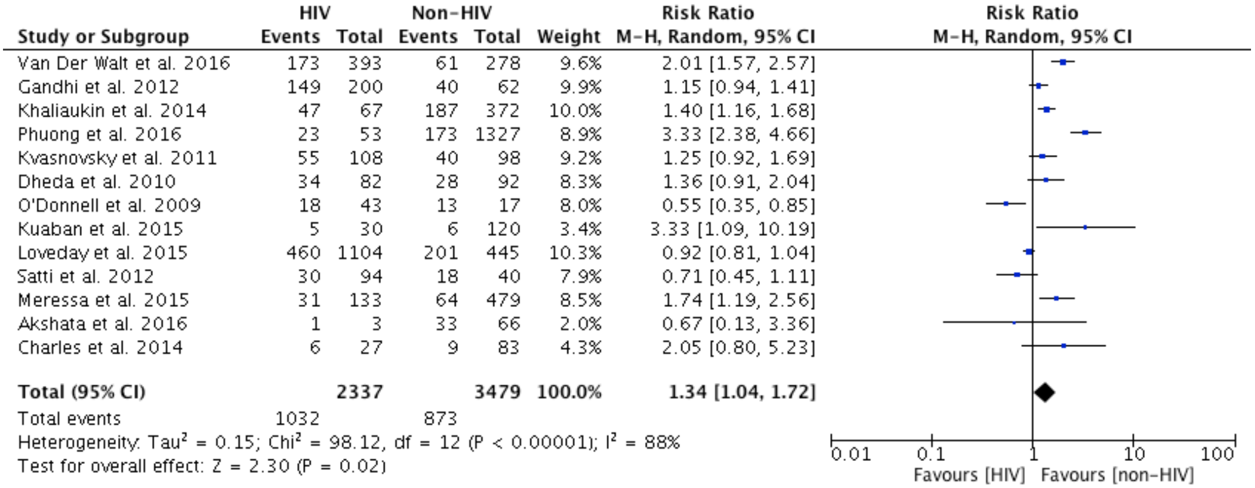

Supp. Fig. 14: Relative Risk of the primary outcome in MDR/XDRTB comparing those with and without HIV from lowest to highest ART use.

Study or Subgroup	Smokers Events	Smokers Total	Non-Smokers Events	Non-Smokers Total	Weight	Risk Ratio M-H, Random, 95% CI
Kurbatova et al. 2012	84	272	526	1496	12.3%	0.88 [0.73, 1.06]
Pazarli et al. 2013	11	61	3	42	2.9%	2.52 [0.75, 8.51]
Duraisamy et al. 2014	16	25	50	154	10.0%	1.97 [1.36, 2.86]
Gegia et al 2012	73	152	106	228	12.1%	1.03 [0.83, 1.28]
Lalor et al. 2013	21	76	251	634	10.0%	0.70 [0.48, 1.02]
Ahmad et al. 2015	7	32	38	164	6.0%	0.94 [0.46, 1.92]
Vashakidze et al. 2013	4	27	13	48	3.8%	0.55 [0.20, 1.51]
Jain et al. 2014	32	47	40	83	11.0%	1.41 [1.05, 1.90]
Cegielski et al. 2016	66	268	417	976	12.0%	0.58 [0.46, 0.72]
Dheda et al. 2010	10	49	52	125	7.2%	0.49 [0.27, 0.89]
Tang et al. 2013	95	165	239	421	12.7%	1.01 [0.87, 1.18]
**Total (95% CI)**		**1174**		**4371**	**100.0%**	**0.94 [0.75, 1.19]**
Total events	419		1735			

Heterogeneity: $Tau^2 = 0.11$; $Chi^2 = 57.50$, $df = 10$ ($P < 0.00001$); $I^2 = 83\%$

Test for overall effect: $Z = 0.48$ ($P = 0.63$)

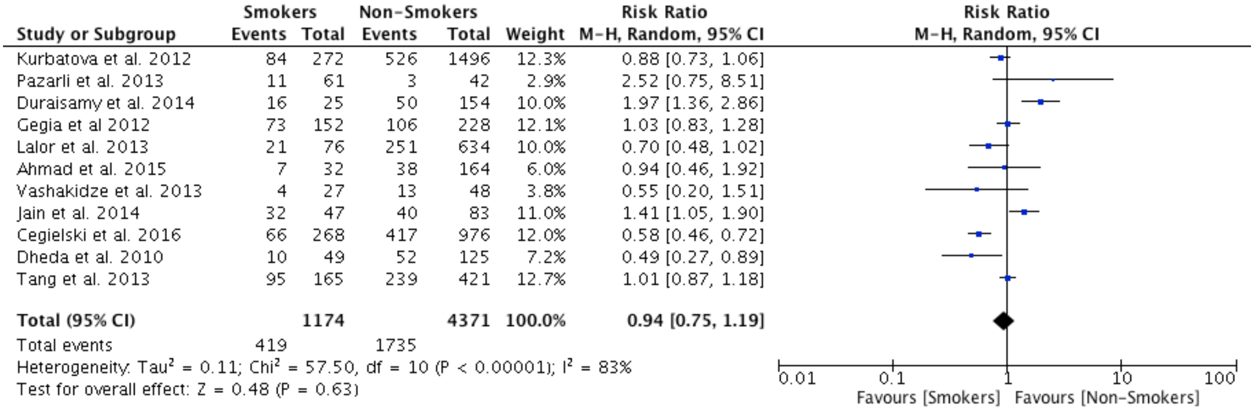

Supp. Fig. 15: Relative Risk of the primary outcome in MDR/XDRTB comparing smokers and non-smokers from low to high quality study.

Study or Subgroup	Smokers Events	Smokers Total	Non-Smokers Events	Non-Smokers Total	Weight	Risk Ratio M-H, Random, 95% CI
Dheda et al. 2010	10	49	52	125	11.9%	0.49 [0.27, 0.89]
Gegia et al 2012	73	152	106	228	28.2%	1.03 [0.83, 1.28]
Lalor et al. 2013	21	76	251	634	19.7%	0.70 [0.48, 1.02]
Pazarli et al. 2013	11	61	3	42	3.8%	2.52 [0.75, 8.51]
Tang et al. 2013	95	165	239	421	31.4%	1.01 [0.87, 1.18]
Vashakidze et al. 2013	4	27	13	48	5.1%	0.55 [0.20, 1.51]
**Total (95% CI)**		**530**		**1498**	**100.0%**	**0.87 [0.68, 1.12]**
Total events	214		664			

Heterogeneity: Tau² = 0.05; Chi² = 12.70, df = 5 (P = 0.03); I² = 61%

Test for overall effect: Z = 1.09 (P = 0.28)

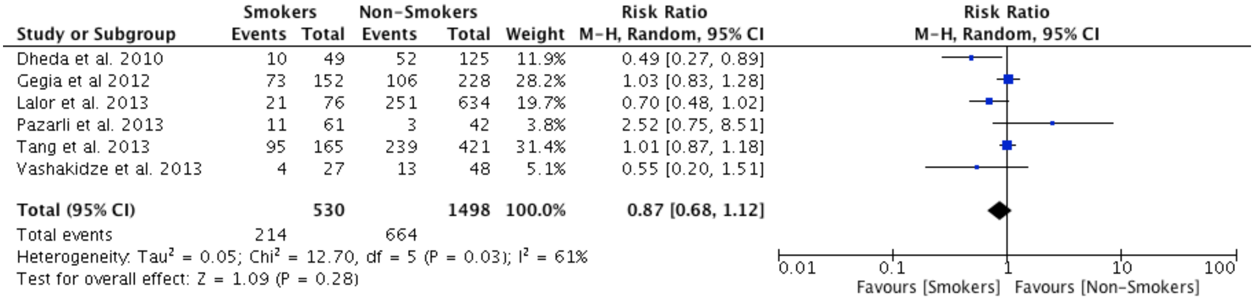

Supp. Fig. 16: Relative Risk of the primary outcome in MDR/XDRTB comparing smokers and non-smokers in high GDP countries.

Study or Subgroup	Smokers Events	Smokers Total	Non-Smokers Events	Non-Smokers Total	Weight	Risk Ratio M-H, Random, 95% CI
Ahmad et al. 2015	7	32	38	164	20.3%	0.94 [0.46, 1.92]
Duraisamy et al. 2014	10	49	52	125	22.9%	0.49 [0.27, 0.89]
Jain et al. 2014	32	47	40	83	29.2%	1.41 [1.05, 1.90]
Lalor et al. 2013	21	76	251	634	27.6%	0.70 [0.48, 1.02]
**Total (95% CI)**		**204**		**1006**	**100.0%**	**0.84 [0.50, 1.42]**
Total events	70		381			

Heterogeneity: $Tau^2 = 0.23$; $Chi^2 = 16.01$, df = 3 (P = 0.001); $I^2 = 81\%$
Test for overall effect: Z = 0.64 (P = 0.52)

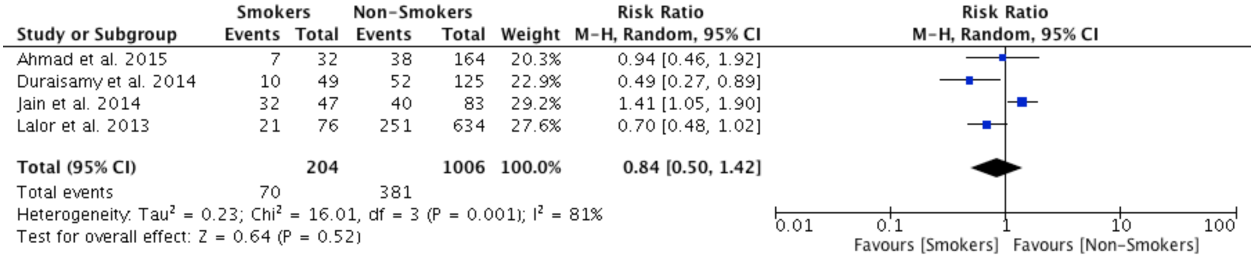

Supp. Fig. 17: Relative Risk of the primary outcome in MDR/XDRTB comparing smokers and non-smokers in low GDP countries.

Study or Subgroup	AM Events	AM Total	Non-AM Events	Non-AM Total	Weight	Risk Ratio M-H, Random, 95% CI
Kurbatova et al. 2012	207	489	352	1279	9.1%	1.54 [1.34, 1.76]
Leimane et al. 2005	48	125	21	79	6.3%	1.44 [0.94, 2.22]
Bendayan et al. 2011	15	34	47	101	6.2%	0.95 [0.62, 1.46]
Cox et al. 2007	10	20	23	67	5.1%	1.46 [0.84, 2.52]
Duraisamy et al. 2014	14	16	52	163	7.7%	2.74 [2.05, 3.67]
Gegia et al 2012	51	94	128	286	8.4%	1.21 [0.97, 1.52]
Lalor et al. 2013	27	90	245	620	7.3%	0.76 [0.55, 1.06]
Franke et al. 2008	9	28	58	643	4.8%	3.56 [1.97, 6.44]
Jain et al. 2014	22	27	50	103	8.0%	1.68 [1.28, 2.19]
Shin et al. 2006	26	86	30	158	6.0%	1.59 [1.01, 2.51]
Vashakidze et al. 2013	1	11	16	64	0.8%	0.36 [0.05, 2.47]
Cegielski et al. 2016	83	179	400	1065	8.8%	1.23 [1.04, 1.47]
Kuksa et al. 2014	36	64	27	69	6.9%	1.44 [1.00, 2.07]
Miller et al. 2012	126	253	34	154	7.4%	2.26 [1.64, 3.11]
Oliveira et al. 2013	22	61	116	303	7.0%	0.94 [0.66, 1.35]
**Total (95% CI)**		**1577**		**5154**	**100.0%**	**1.45 [1.21, 1.74]**
Total events	697		1599			

Heterogeneity: $Tau^2 = 0.09$; $Chi^2 = 69.14$, df = 14 ($P < 0.00001$); $I^2 = 80\%$
Test for overall effect: Z = 4.03 ($P < 0.0001$)

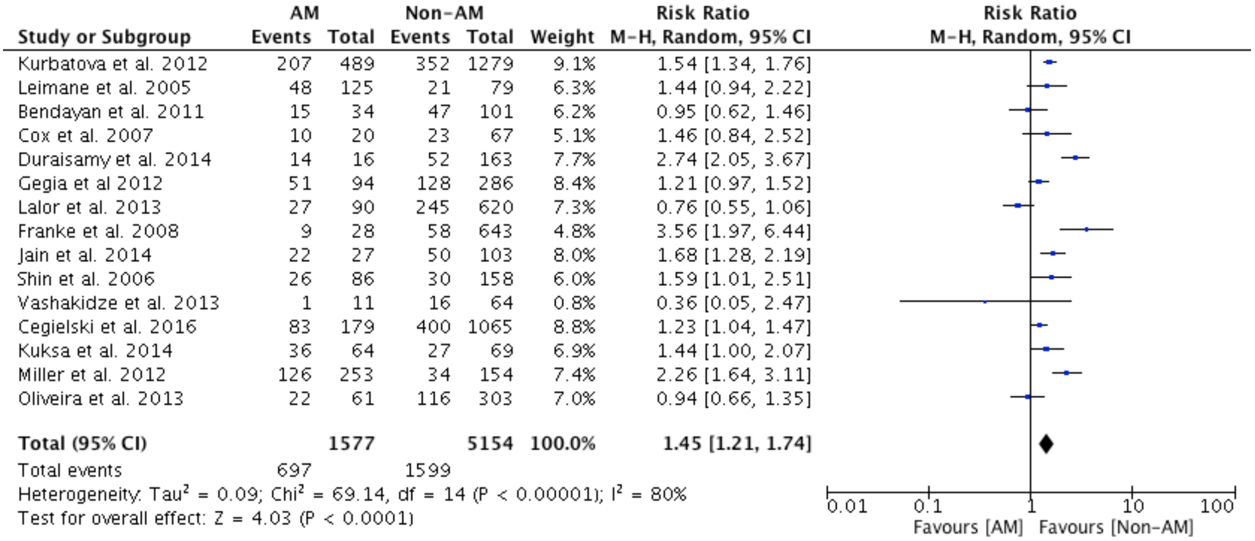

Supp. Fig. 18: Relative Risk of the primary outcome in MDR/XDRTB in those with alcohol misuse (AM) compared to those without alcohol misuse from low to high study quality.

	AM		Non-AM			Risk Ratio
Study or Subgroup	Events	Total	Events	Total	Weight	M-H, Random, 95% CI
Bendayan et al. 2011	15	34	47	101	11.7%	0.95 [0.62, 1.46]
Franke et al. 2008	9	28	58	643	9.2%	3.56 [1.97, 6.44]
Gegia et al 2012	51	94	128	286	15.1%	1.21 [0.97, 1.52]
Kuksa et al. 2014	36	64	27	69	12.8%	1.44 [1.00, 2.07]
Leimane et al. 2005	48	125	21	79	11.7%	1.44 [0.94, 2.22]
Miller et al. 2012	126	253	34	154	13.6%	2.26 [1.64, 3.11]
Oliveira et al. 2013	22	61	116	303	12.9%	0.94 [0.66, 1.35]
Shin et al. 2006	26	86	30	158	11.3%	1.59 [1.01, 2.51]
Vashakidze et al. 2013	1	11	16	64	1.7%	0.36 [0.05, 2.47]
**Total (95% CI)**		**756**		**1857**	**100.0%**	**1.44 [1.11, 1.88]**
Total events	334		477			

Heterogeneity: Tau^2 = 0.11; Chi^2 = 29.65, df = 8 (P = 0.0002); I^2 = 73%
Test for overall effect: Z = 2.72 (P = 0.007)

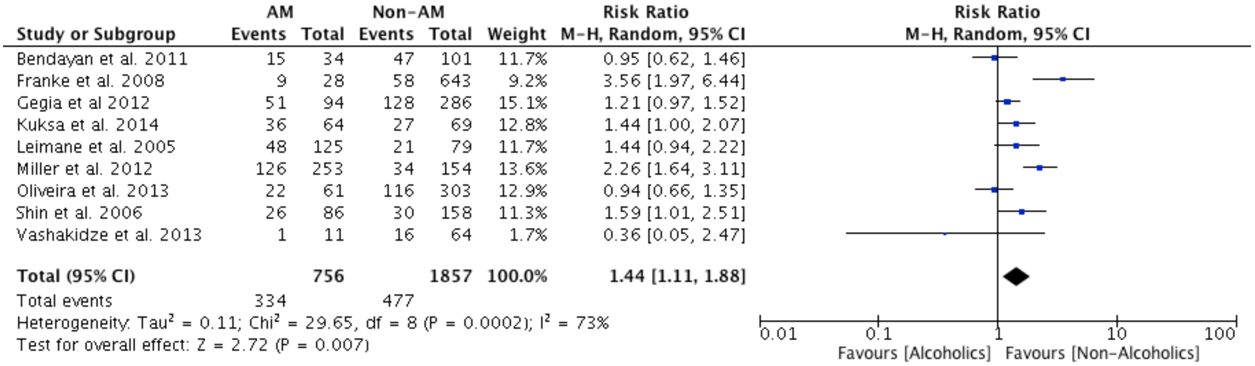

Supp. Fig. 19: Relative Risk of the primary outcome in MDR/XDRTB in those with alcohol misuse (AM) compared to those without alcohol misuse in high GDP countries.

Study or Subgroup	AM Events	AM Total	Non-AM Events	Non-AM Total	Weight	Risk Ratio M-H, Random, 95% CI
Cox et al. 2007	10	20	23	67	22.8%	1.46 [0.84, 2.52]
Duraisamy et al. 2014	14	16	52	163	25.8%	2.74 [2.05, 3.67]
Jain et al. 2014	22	27	50	103	26.0%	1.68 [1.28, 2.19]
Lalor et al. 2013	27	90	245	620	25.4%	0.76 [0.55, 1.06]
**Total (95% CI)**		**153**		**953**	**100.0%**	**1.51 [0.79, 2.89]**
Total events	73		370			

Heterogeneity: $Tau^2 = 0.41$; $Chi^2 = 45.17$, $df = 3$ ($P < 0.00001$); $I^2 = 93\%$

Test for overall effect: $Z = 1.23$ ($P = 0.22$)

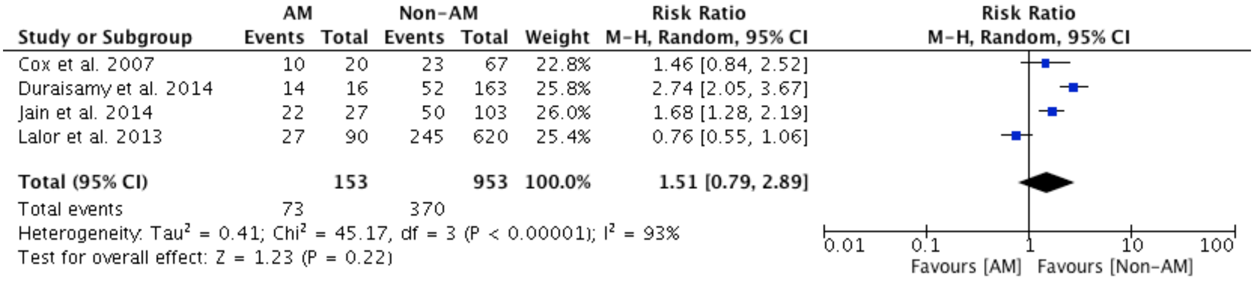

Supp. Fig. 20: Relative Risk of the primary outcome in MDR/XDRTB in those with alcohol misuse (AM) compared to those without alcohol misuse in low GDP countries.

	Data Missing (default/data unaccounted for/lost to follow up/transfer out)
**Akshata *et al.* 2016 [82]**	N/A
**Bendayan *et al.* 2011 [63]**	N/A
**Cegielski *et al.* 2016 [14]**	0.19
**Gegia *et al.* 2012 [45]**	N/A
**Gler *et al.* 2013 [80]**	N/A
**Jeon *et al.* 2009 [73]**	0.23
**Kempker *et al.* 2015 [44]**	N/A
**Kuksa *et al.* 2014 (XDR data only) [60]**	N/A
**Kurbatova *et al.* 2012 [68]**	0.12
**Kwak *et al.* 2015 [10]**	N/A
**Leimane *et al.* 2005 [28]**	N/A

Supplemental Table 1: Proportion of data missing amongst participants with DM.

	**Data Missing (default/data unaccounted for/lost to follow up/transfer out)**
**Park *et al.* 1996 [15]**	0.16
**Shah *et al.* 2008 [49]**	0.03
**Eker *et al.* 2008 [61]**	N/A
**O'Donnell *et al.* 2009 [55]**	N/A
**Brust *et al.* 2010 [53]**	0.75
**Dheda *et al.* 2010 [13]**	N/A
**Farley *et al.* 2011 [64]**	0.21
**Kvasnovsky *et al.* 2011 [48]**	N/A
**Bendayan *et al.* 2011 [63]**	N/A
**Kurbatova *et al.* 2012 [68]**	0.30
**Seddon *et al.* 2012 [11]**	N/A
**Satti *et al.* 2012 [65]**	0.02
**Gegia *et al.* 2012 [45]**	N/A
**Gandhi *et al.* 2012 [72]**	N/A
**Oliveira *et al.* 2013 [69]**	N/A
**Kuksa *et al.* 2014 [60]**	N/A
**Dolgusev *et al.* 2014 [66]**	N/A
**Hicks *et al.* 2014 [57]**	N/A
**Charles *et al.* 2014 [76]**	0.04
**Chung-Delgado *et al.* 2014 [81]**	N/A
**Khaliaukin *et al.* 2014 [67]**	0.03
**Elliott *et al.* 2014 [50]**	N/A
**Duraisamy *et al.* 2014 [12]**	N/A
**Marais *et al.* 2014 [78]**	0.26
**Oladimeji *et al.* 2014 [56]**	N/A
**Kuaban *et al.* 2015 [52]**	N/A
**Meressa *et al.* 2015 [43]**	0.07
**Kempker *et al.* 2015 [44]**	N/A
**Van Altena *et al. 2016 [54]***	0.14
**Akshata *et al.* 2016 [82]**	N/A
**Phuong *et al.* 2016 [59]**	N/A

Supplemental Table 2: Proportion of data missing amongst participants with HIV.

	Data Missing (default/data unaccounted for/lost to follow up/transfer out)
**Ahmad *et al*. 2015 [58]**	0.03
**Cegielski *et al*. 2016 [14]**	0.21
**Dheda *et al*. 2010 [13]**	N/A
**Duraisamy *et al*. 2014 [12]**	N/A
**Gegia *et al*. 2012 [45]**	N/A
**Kurbatova *et al*. 2012 [68]**	0.11
**Lalor *et al*. 2013 [71]**	0.28
**Pazarli *et al*. 2013 [74]**	N/A
**Tang *et al*. 2013 [34]**	N/A
**Vashakidze *et al*. 2013 [51]**	N/A

Supplemental Table 3: Proportion of data missing amongst participants that smoke.

	Data Missing (default/data unaccounted for/lost to follow up/transfer out)
**Bendayan *et al.* 2011 [63]**	N/A
**Cegielski *et al.* 2016 [14]**	0.22
**Cox *et al.* 2007 [62]**	0.35
**Duraisamy *et al.* 2014 [12]**	N/A
**Franke *et al.* 2008 [70]**	0.32
**Gegia *et al.* 2012 [45]**	N/A
**Jain *et al.* 2014 [75]**	N/A
**Kuksa *et al.* 2014 [60]**	N/A
**Kurbatova *et al.* 2012 [68]**	0.22
**Lalor *et al.* 2013 [71]**	0.30
**Leimane *et al.* 2005 [28]**	0.15
**Miller *et al.* 2012 [46]**	0.29
**Oliveira *et al.* 2013 [69]**	N/A
**Shin *et al.* 2006 [77]**	N/A
**Vashakidze *et al.* 2013 [51]**	N/A

Supplemental Table 4: Proportion of data missing amongst participants with alcohol misuse.

	**ART proportion**
**Park *et al.* 1996 [15]**	1
**Shah *et al.* 2008 [49]**	N/A
**Eker *et al.* 2008 [61]**	N/A
**O'Donnell *et al.* 2009 [55]**	N/A
**Brust *et al.* 2010 [53]**	1 (44% started pre TB tx)
**Dheda *et al.* 2010 [13]**	N/A
**Farley *et al.* 2011 [64]**	0.63
**Kvasnovsky *et al.* 2011 [48]**	N/A
**Bendayan *et al.* 2011 [63]**	N/A
**Kurbatova *et al.* 2012 [68]**	N/A
**Seddon *et al.* 2012 [11]**	N/A
**Satti *et al.* 2012 [65]**	N/A
**Gegia *et al.* 2012 [45]**	0.28
**Gandhi *et al.* 2012 [72]**	N/A
**Oliveira *et al.* 2013 [69]**	N/A
**Kuksa *et al.* 2014 [60]**	N/A
**Dolgusev *et al.* 2014 [66]**	0.40
**Hicks *et al.* 2014 [57]**	0.73
**Charles *et al.* 2014 [76]**	N/A
**Chung-Delgado *et al.* 2014 [81]**	N/A
**Khaliaukin *et al.* 2014 [67]**	0.55
**Elliott *et al.* 2014 [50]**	0.86
**Duraisamy *et al.* 2014 [12]**	N/A
**Marais *et al.* 2014 [78]**	0.98
**Oladimeji *et al.* 2014 [56]**	0.67
**Kuaban *et al.* 2015 [52]**	N/A
**Meressa *et al.* 2015 [43]**	N/A
**Kempker *et al.* 2015 [44]**	N/A
**Van Altena *et al.* *2016 [54]***	0.53
**Akshata *et al.* 2016 [82]**	0.96
**Phuong *et al.* 2016 [59]**	N/A
**Park *et al.* 1996 [15]**	N/A
**Shah *et al.* 2008 [49]**	N/A
**Eker *et al.* 2008 [61]**	0.24

Supplemental Table 5: Proportion of participants with HIV on ART.

	**Current vs ever smoker**
**Ahmad *et al*. 2015 [58]**	Ever
**Cegielski *et al*. 2016 [14]**	Ever
**Dheda *et al*. 2010 [13]**	Ever
**Duraisamy *et al*. 2014 [12]**	Ever
**Gegia *et al*. 2012 [45]**	Current
**Kurbatova *et al*. 2012 [68]**	Ever
**Lalor *et al*. 2013 [71]**	Ever
**Pazarli *et al*. 2013 [74]**	Ever
**Tang *et al*. 2013 [34]**	Ever
**Vashakidze *et al*. 2013 [51]**	Current

Supplemental Table 6: Definition of smoker used in study.

Diabetics	Unadjusted Effect Estimate	Adjusted Effect Estimate
**Chung-Delgado *et al.* 2015 [32]**	Hazard Ratio (HR): 4.1 (95%CI:2.15-7.85)	HR: 5.42 (95%CI:2.66-11.04)
**Kendall *et al.* 2013 [26]**	HR: 0.36<br><br>(95%CI:0.05-2.69)	N/A
**Leimane *et al.* 2005 [28]**	HR: 2.5<br><br>(95%CI:0.7-8.5)	N/A
**Liu *et al.* 2011 [27]**	OR: 0.65 (95%CI:0.37-1.14)-MDR data only	OR: 0.73 (95%CI:0.38-1.43) MDR data only
**Tang *et al.* 2013 [34]**	N/A	OR: 0.305 (95%CI: 0.140-0.663)

Supplemental Table 7: Effect estimates for combined negative treatment outcomes in participants with DM from studies that did not provide raw data.

HIV	Unadjusted Effect Estimate	Adjusted Effect Estimate
**Mitnick *et al*. 2013 [25]**	HR: 3.16 (95%CI: 1.29-7.74)	HR: 2.72 (95%CI: 1.03-7.24)
**Pietersen *et al*. 2015 [30]**	N/A	OR: 2.9 (95%CI: 1.34-6.3)
**Kendall *et al*. 2013 [26]**	HR: 0.61 (95%CI: 0.32-1.14)	N/A
**Seung *et al*. 2009 [29]**	OR: 1.9 (95%CI: 0.65-6.5)	N/A
**Velasquez *et al*. 2014 [31]**	HR: 1.7 (95%CI: 0.41-7.03)	HR: 3.4 (95%CI: 0.72-16.2)
**Chung-Delgado *et al*. 2015 [32]**	HR: 2.33 (95%CI: 0.83-6.51)	HR: 3.18 (95%CI: 1.05-9.69)
**Kliiman *et al*. 2009 [33]**	N/A	OR: 10.16 (95%CI: 1.17-88.4)

Supplemental Table 8: Effect estimates for combined negative treatment outcomes in participants with HIV from studies that did not provide raw data.

Smokers	Unadjusted Effect Estimate	Adjusted Effect Estimate
**Kendall *et al*. 2013 [26]**	HR: 2.02 (95%CI: 1.01-4.05)	N/A
**Chung-Delgado *et al*. 2015 [32]**	HR: 0.4 (95%CI: 0.12-1.29)	N/A

Supplemental Table 9: Effect estimates for combined negative treatment outcomes in participants that smoked from studies that did not provide raw data.

Alcohol Misuse	Unadjusted Effect Estimate	Adjusted Effect Estimate
**Kendall *et al*. 2013 [26]**	HR: 2.24<br>(95%CI: 1.22-4.13)	HR: 2.11<br>(95%CI: 1.11-4.02)
**Chung-Delgado *et al*. 2015 [32]**	N/A	HR: 0.51<br>(95%CI: 0.21-1.20)
**Kliiman *et al*. 2009 [33]**	OR: 2.42<br>(95%CI: 1.34-4.37)	OR: 1.94<br>(95%CI: 0.96-3.92)

Supplemental Table 10: Effect estimates combined negative treatment outcomes in participants with alcohol misuse from studies that did not provide raw
